# Knocking out *Fkbp51* decreases CCl_4_-induced liver injury through enhancement of mitochondrial function and Parkin activity

**DOI:** 10.1186/s13578-023-01184-3

**Published:** 2024-01-02

**Authors:** Bin Qiu, Zhaohui Zhong, Longyu Dou, Yuxue Xu, Yi Zou, Korri Weldon, Jun Wang, Lingling Zhang, Ming Liu, Kent E. Williams, John Paul Spence, Richard L. Bell, Zhao Lai, Weidong Yong, Tiebing Liang

**Affiliations:** 1 Department of Medicine, Indiana University, School of Medicine, Indianapolis, 46202 USA; 2 Department of Surgery, Indiana University, School of Medicine, Indianapolis, 46202 USA; 3 Department of Pediatrics, Indiana University, School of Medicine, Indianapolis, 46202 USA; 4 Department of Psychiatry, Indiana University, School of Medicine, Indianapolis, 46202 USA; 5 Institute of Laboratory Animal Science, Chinese Academy of Medical Sciences & Peking Union Medical College, Beijing, 100021 China; 6 General Surgery Department, Peking University People’s Hospital, Beijing, 100032 China; 7 Department of Pharmacology, Yale University School of Medicine, New Haven, CI 06520 USA; 8 Greehey Children’s Cancer Research Institute, UT Health, San Antonio, TX 78229 USA

**Keywords:** *FK506binding protein 5/Fkbp51*, RNA-seq, Mitochondrial quality control (MQC), SAFit2, Liver disease treatment

## Abstract

**Background and aims:**

Previously, we found that FK506 binding protein 51 (*Fkbp51*) knockout (KO) mice resist high fat diet-induced fatty liver and alcohol-induced liver injury. The aim of this research is to identify the mechanism of *Fkbp51* in liver injury.

**Methods:**

Carbon tetrachloride (CCl_4_)-induced liver injury was compared between *Fkbp51* KO and wild type (WT) mice. Step-wise and in-depth analyses were applied, including liver histology, biochemistry, RNA-Seq, mitochondrial respiration, electron microscopy, and molecular assessments. The selective FKBP51 inhibitor (SAFit2) was tested as a potential treatment to ameliorate liver injury.

**Results:**

*Fkbp51* knockout mice exhibited protection against liver injury, as evidenced by liver histology, reduced fibrosis-associated markers and lower serum liver enzyme levels. RNA-seq identified differentially expressed genes and involved pathways, such as fibrogenesis, inflammation, mitochondria, and oxidative metabolism pathways and predicted the interaction of FKBP51, Parkin, and HSP90. Cellular studies supported co-localization of Parkin and FKBP51 in the mitochondrial network, and Parkin was shown to be expressed higher in the liver of KO mice at baseline and after liver injury relative to WT. Further functional analysis identified that KO mice exhibited increased ATP production and enhanced mitochondrial respiration. KO mice have increased mitochondrial size, increased autophagy/mitophagy and mitochondrial-derived vesicles (MDV), and reduced reactive oxygen species (ROS) production, which supports enhancement of mitochondrial quality control (MQC). Application of SAFit2, an FKBP51 inhibitor, reduced the effects of CCl_4_-induced liver injury and was associated with increased Parkin, pAKT, and ATP production.

**Conclusions:**

Downregulation of FKBP51 represents a promising therapeutic target for liver disease treatment.

**Graphical Abstract:**

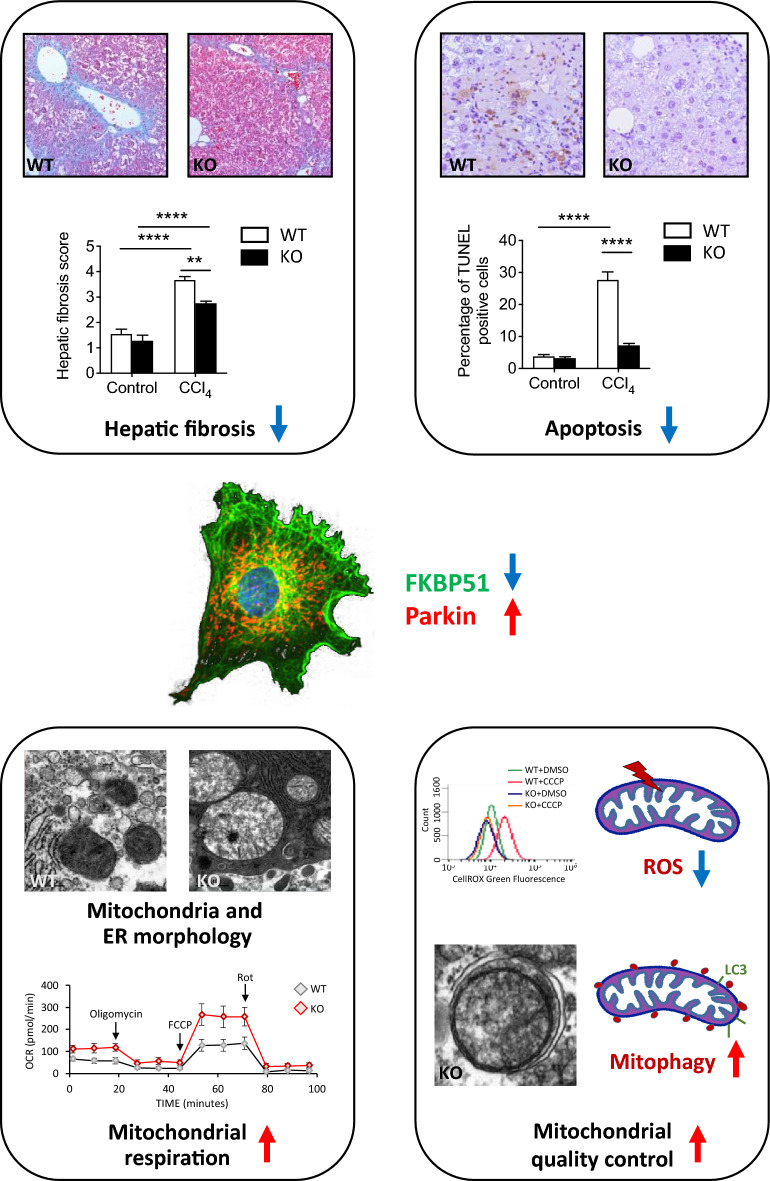

**Supplementary Information:**

The online version contains supplementary material available at 10.1186/s13578-023-01184-3.

## Introduction

Liver diseases are chronic conditions, affecting more than 100 million people worldwide, which can result from obesity, alcohol consumption, viral infection, and toxic-metabolic insults [1]. The subsequent development of cirrhosis increases the risk of hepatocellular carcinoma (HCC), and no effective treatments currently exist to reverse advanced liver disease-associated phenotypes. Thus, early interventions to reduce liver damage and halt further liver disease progression are necessary. As patients with liver disease often present with dysfunction in mitochondrial ultrastructure, dynamics, activity in respiratory chain complexes, and adenosine triphosphate (ATP) synthesis, improving mitochondrial function is one promising strategy to treat chronic liver diseases [2–4].

FK506 binding protein 51 (FKBP51), well known as a glucocorticoid receptor (GR) binding protein, is an essential chaperone of heat-shock protein 90 (HSP90) that affects hypothalamus–pituitary–adrenal (HPA)-axis function in response to stress, regulates protein post-translational modification, and participates in the function of protein signaling pathways [5–9]. Previous studies have shown that FKBP51 plays key roles in metabolic function regulation [10–12]. We have shown that high fat diet-fed *Fkbp51* knockout (KO) mice are resistant to weight gain, hepatic steatosis, and adiposity, and that these effects are correlated with increased energy expenditure [13]. FKBP51 also acts as a chaperone of Akt and affects GRα and PPARγ phosphorylation, and its ablation reduces lipid accumulation and decreases the expression of adipogenic genes [14, 15]. In vitro modeling of hypoxic stress demonstrated that loss of FKBP51 inhibits adipocyte differentiation [16], and others found that maternal nutrient restriction increases the signaling of hypoxia-inducible pathways, including FKBP51, in fetal mouse liver [17]. Furthermore, the loss of *Fkbp51* was demonstrated to ameliorate alcohol-induced liver injury [18]. FKBP51 plays critical roles in hormone nuclear translocation and protein folding [19]. Studies have demonstrated co-localization of FKBP51 with mitochondria and have noted nuclear-mitochondrial shuttling of the protein; a mechanism triggered in response to oxidative stress, adipogenesis, and viral infection [9, 20, 21]. Mitochondrial reactive oxygen species (ROS)-mediated metabolic pathway alterations, and higher levels of ROS generally are known to enhance liver disease progression [11, 15, 22, 23]. However, the role of FKBP51 in mitochondrial function in response to drug-induced liver injury has not been fully elucidated.

Mitochondrial quality control (MQC) is vital in pathophysiological states, supporting nutrient recycling, toxin degradation, and energy regeneration, and its dysfunction is a feature of both alcoholic liver disease (ALD) and non-alcoholic fatty liver disease (NAFLD) [24]. MQC maintains mitochondrial structural integrity, including the removal of portions of damaged mitochondria through mitochondrial-derived vesicles (MDVs), and regulates mitochondrial biogenesis through fission and fusion [25, 26]. Autophagy is another mechanism employed to adapt to liver injury induced by carbon tetrachloride (CCl_4_) [27, 28]. A network of proteases and chaperones are involved at different molecular levels of MQC. One well-studied participant in MQC is Parkin, which functions in regulating misfolded protein degradation via the ubiquitin–proteasome system, the shedding of vesicles via interaction with PTEN Induced Kinase 1 (PINK1) [29], and mitophagy through triggering the polyubiquitination of mitochondrial membrane proteins and proteasomal activation [26].

In this study, we applied the widely used CCl_4_-induced liver injury model with modification [30, 31] and showed that *Fkbp51* KO mice were protected against liver injury as evidenced by liver histology, fibrogenesis markers, and serum liver enzymes. RNA-seq pointed to the enrichment of mitochondrial function-related pathways, particularly an increase in Parkin expression. We further examined mitochondrial morphology, ATP production, and MQC-related changes, and revealed that loss of FKBP51 enhances mitochondrial function. Application of SAFit2, a selective inhibitor of FKBP51, demonstrated the potential of targeting FKBP51 in liver injury treatment. Thus, downregulation of FKBP51 activity and a subsequent increase in Parkin and MQC activity is a potential therapeutic approach for the treatment of liver disease. These findings shed new light on FKBP51 with strong implications for its role in the regulation of liver damage via Parkin-associated mitochondrial regulation.

## Results

### Elimination of ***Fkbp51*** ameliorates CCl_4_-induced liver injury

**Fig. 1:**
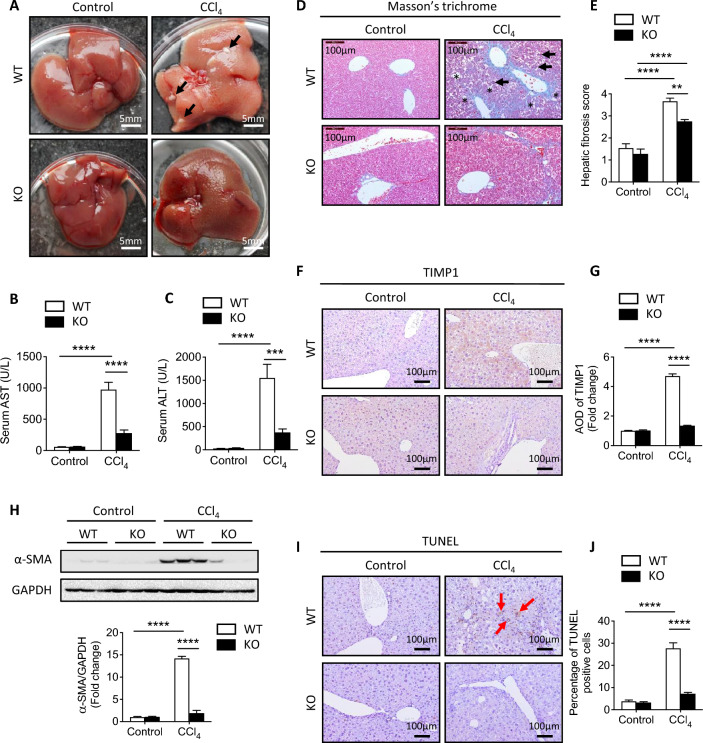
Ablation of *Fkbp51* gene reduces CCl_4_-induced liver damage. **A** Gross liver structure was observed in control and CCl_4_-treated *Fkbp51* KO and WT littermate mice. In WT liver with CCl_4_ treatment, the fibrosis nodules are indicated by black arrows. **B**, **C** Serum analyses of Control and CCl_4_-treated *Fkbp51* KO and WT were performed to determine the concentrations of AST and ALT. **D** Histological examination of Control and CCl_4_-treated *Fkbp51* KO and WT liver sections stained with Masson’s trichrome. Mice treated with CCl_4_ displayed portal vein fibrosis and ballooning (star), and hepatocytes undergoing necrosis (arrow). **E** CCl_4_-treated WT liver sections possess significantly higher hepatic fibrosis scores as compared to *Fkbp51* KO liver. **F** Expression patterns of TIMP1 detected by IHC are higher in the liver sections of CCl_4_-treated WT mice relative to KO mice. **G** Quantitation of AOD of TIMP1 in liver sections. **H**. Immunoblot and quantification showed less α-SMA protein in CCl_4_-treated KO than WT. **I** TUNEL was performed to measure apoptosis in the liver. More apoptotic cells (indicated by arrows) were found following CCl_4_ treatment in WT than KO liver. **J** Quantitation of percentage TUNEL-positive hepatocytes (%) in liver sections. Graphs represent mean values ± SEM from 10 mice for each group. *p* values were determined by two-way ANOVA with the statistical significance labeled as follows: ** as *p* < 0.01, *** as *p* < 0.001 and **** as *p* < 0.0001. Key: WT, wild type; KO, *Fkbp51* KO; AOD, average optical density

In order to investigate the involvement of FKBP51 in liver injury, wildtype (WT) and knockout (KO) mice were subjected to CCl_4_ treatment three times per week for a duration of 2 weeks. For the purpose of comparing and analyzing the gross liver phenotype among the groups, the livers were removed from the mice without undergoing perfusion. In the WT mouse liver, CCl_4_ treatment resulted in a robust color change, rough surface, and stiffness with multiple fibrotic nodules (Fig. 1a, arrows). However, the CCl_4_-treated livers of KO mice exhibited fewer of these pathophysiological phenotypes, suggesting a protective effect of *Fkbp51* KO (Fig. 1a). Serum levels of aspartate transaminase (AST) and alanine aminotransferase (ALT) (Fig. 1b, c), which were low at baseline, were significantly more elevated in WT mice than in KO following CCl_4_ treatment (Fig. 1b, c), indicating less liver damage in the KO mouse group.

Masson’s trichrome staining (Fig. 1d) was used to evaluate histopathologic alterations in the tissue. Both WT and KO livers in the control group contained cells possessing a normal morphology and regular collagen distribution. After CCl_4_ treatment however, ballooning (*), hepatocellular necrosis (arrows), and extensive collagen deposition, were observed in WT mice (Fig. 1d, CCl_4_ panel). Notably, collagen bundles surrounding hepatic nodules were significantly attenuated in KO mice when compared to WT mice (Fig. 1d). The grade of liver fibrosis was scored following published guidelines and was shown to be significantly lower in KO mice relative to WT mice following CCl_4_ treatment (*P* < 0.0001) with no differences observed at baseline (Fig. 1e) [32]. Consistent with these findings, immunohistochemical (IHC) labeling showed that hepatic collagen I deposition was much lower in KO relative to WT mice after CCl_4_ treatment (Additional file 2: Fig. S1A). Other fibrogenesis-associated markers upregulated to a greater degree in WT mice after CCl_4_ injection included tissue inhibitor of metalloproteinase 1 (TIMP-1) (Fig. 1f, g) and connective tissue growth factor (CTGF) (Additional file 2: Fig. S1B). Alpha smooth muscle actin (α-SMA), a marker of hepatic stellate cell (HSC) activation, was also expressed at higher levels in CCl_4_-injured WT liver than in KO as measured by western blotting and IHC labeling (Fig. 1h and Additional file 2: Fig. S1C). These data indicate that KO mice are less susceptible to CCl_4_-induced liver injury-related fibrosis, matrix remodeling, and HSC activation relative to WT mice. Additionally, more apoptotic cells (arrows), a key feature of liver injury, were found in CCl_4_-treated WT mice compared to KO mice as determined by terminal deoxynucleotidyl transferase deoxyuridine triphosphate nick end labeling (TUNEL) (Fig. 1i, j). The observed morphological and cellular maker alterations demonstrate that loss of *Fkbp51* confers protection from hepatic injury.

### RNA transcriptome profiling identifies pathways and genes important to liver and mitochondrial function

**Fig. 2:**
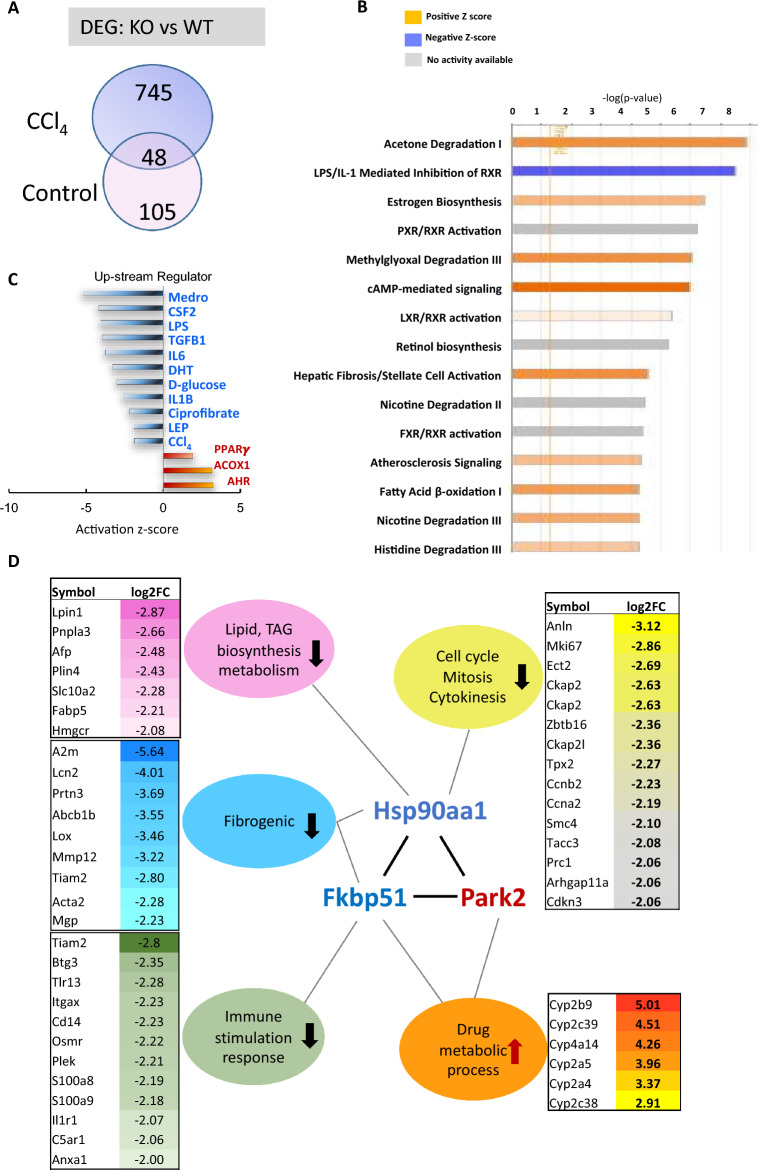
RNA-Seq reveals differentially expressed genes (DEGs), downstream effects, and upstream regulators in Control and CCl_4_-treated *Fkbp51* KO and WT liver samples. **A** Venn Diagram shows differentially expressed genes (DEGs) between CCl_4_-treated *Fkbp51* KO and WT, between vehicle control-treated *Fkbp51* KO and WT, and in common of both conditions. Statistical significance was set as the adjusted *p*-value (adj *p*) < 0.05 and fold change >  = 2. **B** The bar-chart represents downstream effects, and the most significant canonical pathways are plotted by their statistical significance (-log *p*-value) when unique CCl_4_-responsive DEGs served as the input for IPA pathway analysis. **C** Regulator effects analysis featuring the top predicted upstream regulators were plotted by their activation Z-score. Immuno-responsive substances (LPS, TGF-β1, IL-6, and IL-1B), hormone regulators (Medrol, DHT, and LEP), d-glucose, and CCl_4_ are inhibited, while functional enzymes related to fatty acid β-oxidation (ACOX1), ligand-binding receptors (AHR), and nuclear factors (PPARγ) are activated. **D** STRING analysis identified clusters of down- and upregulated genes with log2FC KO/WT higher than 2 involved in several critical functions of liver fibrosis formation. Arrows indicate up- and downregulation and the fold changes are listed. (*Abbreviations*: ACOX1, Acyl-CoA Oxidase 1; AHR, Aryl Hydrocarbon Receptor; DHT, dihydrotestosterone; LEP, leptin; LPS, lipopolysaccharide; Medrol, methylprednisolone. Key: WT, wild type; KO, *Fkbp51* KO

RNA-seq was applied to profile gene expression differences in the livers of KO and WT mice with or without CCl_4_ treatment. Pairwise comparisons identified differentially expressed genes (DEGs). Using a fold change cut off > 2.0 and an adjusted *p*-value (adj *p*) of < 0.05, the number of DEGs were determined between KO and WT for either CCl_4_ injection (745 genes), control solvent (105 genes), or both (48 genes) (Fig. 2a). The 745 unique DEGs between KO and WT for CCl_4_ treatment became our main interest and were used as input for ingenuity pathway analysis (IPA). The bar-chart represents the most significant pathways altered between KO and WT mice after CCl_4_ injury (Fig. 2b). Among those, we observed significant differences in pathways relevant to liver function demonstrating a distinctive response to CCl_4_ injury in *Fkbp51* KO mice. Such pathways included lipopolysaccharides (LPS)/interleukin 1 (IL-1)-mediated inhibition of retinoid X receptor (RXR), hepatic fibrosis/HSC activation, liver X receptor (LXR)/RXR activation, and fatty acid β-oxidation I. Molecules of interest in the IPA pathway are included in Additional file 1: Table S1. Similarly, the identified Kyoto encyclopedia of genes and genomes (KEGG) pathways and involved proteins suggested the PPAR signaling pathway and many others are significantly different between KO and WT mice (Table 1). These signaling pathways directly regulate liver function and are well established in the progression of liver disease pathology, further supporting a critical role of *Fkpb51* in liver injury response [33].

**Table 1:** KEGG pathway analysis has identified significant pathways that are different between KO and WT after CCl_4_-induced liver injury

Pathway description	Observed gene count	FDR	Matching proteins in the data
PPAR signaling pathway	9	7.56E-06	ACSL3,ACSL4,APOA2,APOA5,CYP7A1,CYP8B1,FABP4,FABP5,LPL
Metabolic pathways	27	0.00416	ACSL3,ACSL4,AGXT2L1,ALAS1,ALDH3A2,AMDHD1,ATP6V0C,B3GALT1,CSAD,CYP2E1,CYP7A1,CYP8B1,GCLC,GLUL,HAL,HMGCR,HSD3B2,HSD3B7,HYI,ISYNA1,LPIN1,LPIN2,MOCS1,OAT,RRM2,SPHK2,SQLE
Protein processing in endoplasmic reticulum	9	0.00416	DNAJC3,HSP90AA1,HSP90B1,HSPA5,HYOU1,PDIA3,PDIA4,SSR3,SYVN1
Bile secretion	6	0.00453	ABCG5,ABCG8,CYP7A1,HMGCR,NR0B2,SLC10A2
Amoebiasis	7	0.00506	CD14,COL1A1,COL1A2,COL3A1,COL4A1,COL5A3,IL1R1
ECM-receptor interaction	6	0.00897	COL1A1,COL1A2,COL3A1,COL4A1,COL5A3,SPP1
Protein digestion and absorption	6	0.00897	COL1A1,COL1A2,COL3A1,COL4A1,COL5A3,SLC3A1
Platelet activation	7	0.0104	COL1A1,COL1A2,COL3A1,COL5A3,FGA,FGB,FGG
Primary bile acid biosynthesis	3	0.0181	CYP7A1,CYP8B1,HSD3B7
PI3K-Akt signaling pathway	11	0.0181	CDKN1A,COL1A1,COL1A2,COL3A1,COL4A1,COL5A3,HSP90AA1,HSP90B1,IL6R,NR4A1,SPP1
Glutathione metabolism	4	0.0355	GCLC,GSTA2,GSTM4,RRM2
Transcriptional mis-regulation in cancer	7	0.0355	CD14,CDKN1A,CEBPA,CEBPE,ID2,TMPRSS2,ZBTB16
Glycerolipid metabolism	4	0.047	ALDH3A2,LPIN1,LPIN2,LPL

Regulator effects analysis featuring the top predicted upstream regulators were plotted by their activation Z-score (Fig. 2c). Immuno-responsive substances (LPS, transforming growth factor β1 (TGF-β1), and the inactivation of IL-6 and IL-1B in KO mice indicate a decreased liver inflammatory response (Fig. 2c). We also measured immune factors and consistently found lower levels of interferon γ (IFN-γ), IL-6, TGF-β1, and nuclear factor-κB (NFκB) in KO mice relative to WT mice at baseline (Additional file 2: Fig. S2A–D). Following treatment with CCl_4_, the levels of these factors remained significantly lower in KO mice compared to WT (Additional file 2: Fig. S2A–D). The levels of IL-10 and tumor necrosis factor α (TNF-α) were similar between KO and WT mice at baseline but were lower in KO mice compared to WT mice after CCl_4_ injury (Additional file 2: Fig. S2E, F). Additionally, hormone regulators [Medrol, dihydrotestosterone (DHT), and leptin (LEP)], d-glucose, and CCl_4_ were inactivated, while PPARγ, acyl-coA oxidase 1 (ACOX1), and aryl hydrocarbon receptor (AHR) were activated in KO mice after CCl_4_ injury, suggesting that FKBP51 plays a role in hormone nuclear translocation, metabolism of glucose, and other functions in response to CCl_4_-induced injury (Fig. 2c). It was confirmed that glucocorticoid (GC) and fibroblast growth factor (FGF) levels were lower in KO than WT (S-Fig. 2g, h). Further analysis of downstream effect networks identified the engulfment of myeloid cells, endocytosis, and activation of antigen presenting cells (Table 2). Thus, our data support differential responses between KO and WT mice in the recruitment of myeloid cells to the liver and the secretion of inflammatory cytokines through the innate immune system in response to liver injury.

**Table 2:** Identified disease and function categories and involved regulators when comparing *Fkbp51* KO and WT after CCl_4_-induced liver injury

Top regulator effect networks		
Regulators	Disease and function	Consistency score
Akt, APOE, C5, CSF1, CSF2, Ige, IL1, IL17A, IL1A, IL2, KIT	Engulfment of myeloid cells	43.7
Alpha catenin, AR, CSF2, EGFR, FOXA1, GLIS2, IL17A, IL2	Endocytosis, Engulfment of cells	21.1
ACOX1, CREBBP, CSF2, IFI16, IL17A, IL2, OSM, PRKCA, ROCK2	Arteriosclerosis, Endocytosis, Engulfment of cells	12
Akt, Brd4, CSF2, F2, IL17A, KITLG, PRKCD, TLR4	Activation of antigen presenting cells, Endocytosis	20

The most significant DEGs with fold changes (FC) > 4 (or Log2 FC > 2) are included in Additional file 1: Table S2, and were further analyzed for their protein–protein interactions using the Search Tool for the Retrieval of Interacting Genes (STRING) database. Five robust functional gene hubs centered by *Hsp90aa1, Fkbp51*, and *Park2* (encodes Parkin) were identified. Genes in each cluster with their gene expression log2FC between KO and WT are listed in Fig. 2d. Prominently, a group of Cyp450 family members were upregulated in KO mice, while DEGs related to cell cycle, lipid biosynthesis, fibrogenesis, and immune-function were downregulated (Fig. 2d). Further analysis points to FKBP51 as a key player resulting in unique changes in disease and development, physiological systems, signaling pathways, and toxicity following CCl_4_ injury (Additional file 2: Fig. S3A). GO analysis predicted functional categories altered in KO after CCl_4_ injury (Table 3). Enrichment of biological processes, cellular components, and molecular functions further highlighted functionally-relevant changes between KO and WT mice following liver injury using IPA analysis (Additional file 2: Fig. S3B).

**Table 3:** GO analysis predicted functional alterations in *Fkbp51* KO after CCl_4_-induced liver injury

Function	Category	*p*-value		z-Score
Cellular Movement	Cell movement	3.75E−16	Decreased	− 4.673
Cell-To-Cell Signaling and Interaction	Activation of cells	5.45E−10	Decreased	− 4.262
Cellular Function and Maintenance, Inflammatory Response	Phagocytosis	2.21E−06	Decreased	− 3.891
Inflammatory Response	Immune response of cells	1.28E−07	Decreased	− 3.799
Amino Acid Metabolism, Small Molecule Biochemistry	Metabolism of amino acids	4.33E−07	Increased	2.185
Lipid Metabolism, Small Molecule Biochemistry	Conversion of lipid	5.63E−07	Increased	2.312
Organismal Survival	Morbidity or mortality	1.12E−13	Increased	2.442
Organismal Survival	Organismal death	4.81E−13	Increased	2.514

### Elimination of *Fkbp51* affects mitochondrial function-related genes and capacity for ATP production

**Fig. 3:**
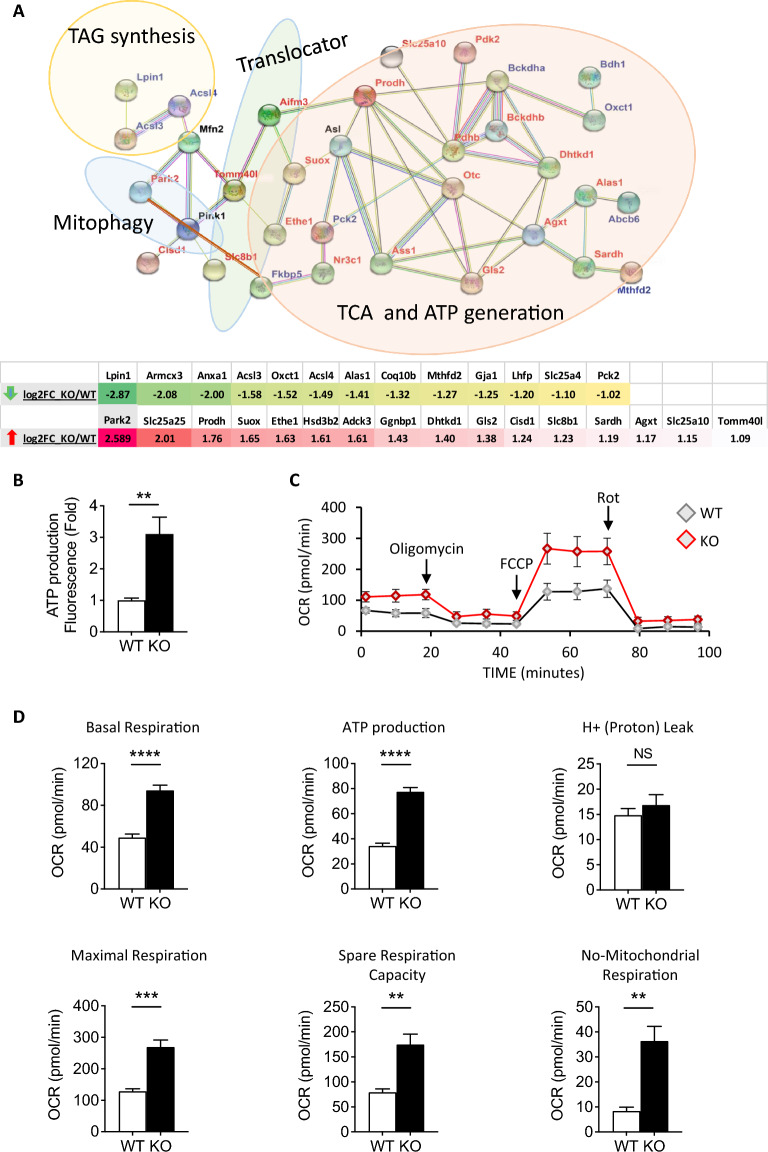
Loss of Fkbp51 increases ATP production by enhanced mitochondrial function. **A** DEG between *Fkbp51* KO and WT after CCl_4_ treatment functionally-related to mitochondria are interconnected based on STRING analysis. Figure was modified from STRING analysis, red-colored gene names indicate upregulation in KO, blue-colored gene names indicate downregulation in KO, and black-colored gene names indicate similar expression between KO and WT (added to identify more interactions). Log2 fold-change (FC) of DEG are included. **B** ATP production of WT and KO MEF cells as determined by PhosphoWork Luminometric ATP Assay. **C** Mitochondrial respiration reflected by OCR was detected in *Fkbp51* KO and WT MEFs during the course of the Seahorse XF Cell Stress Test. The arrow indicates the sequential addition of oligomycin, FCCP, and rotenone (Rot). The OCR profile is normalized to total cultured cell protein. **D** Graph demonstrating increases in KO MEF OCR during basal mitochondrial respiration, ATP production, H^+^ (Proton) leak, maximal respiration, spare respiration capacity, and non-mitochondrial respiration relative to WT. Graphs represent mean values ± SEM from 3 independent experiments. *p* values were determined by student’s unpaired *t*-test with the statistical significance labeled as follows: ** as *p* < 0.01; *** as *p* < 0.001 and **** as *p* < 0.0001; NS, not statistically significant. Key: WT, wild type; KO, *Fkbp51* KO

As *Park2* and *Hsp90* are involved in mitochondrial function, we studied DEGs related to mitochondrial function. We observed that DEGs related to mitochondrial function are overrepresented (more than 10%) (*p* < 0.05, FC > 2) in the data set, and the top DEGs were further analyzed (Fig. 3a). STRING analysis was utilized to cluster the genes based on their functional connections. Genes related to triacylglyceride (TAG) synthesis were downregulated (Lipin 1 (*Lpin1*)*,* acyl-CoA synthetase long chain family member 3 (*Acsl3*)*, and Acsl4*). Translocator genes [translocase of outer mitochondrial membrane 40 like (*Tomm40l*) and solute carrier family 8 member b1 (*Slc8b1*)], and the mitophagy-related gene Parkin (*Park2*) were upregulated in KO following CCl_4_ treatment (Fig. 3a). Additionally, sulfite oxidation-related genes [sulfite oxidase (*Suox*) and ethylmalonic encephalopathy protein 1 (*Ethe1*)] and amino acid degradation-related genes [proline dehydrogenase (*Prodh*), and glutaminase 2 (*Gls2*)] were also upregulated (Fig. 3a). Gene expression fold-changes of these mitochondrial function-related DEGs between KO and WT mice are shown in Fig. 3a. The identification of a functional connection between Parkin and FKBP51 [34], along with the established relationship between Pink1 and FKBP51 [35], strengthens the argument that FKBP51 plays an important role in mitochondrial regulation. Our study further highlights those DEGs with higher FC between KO and WT and their involved functions in mitochondria.

Based on the data demonstrating that mitochondrial function-related DEGs were altered, we compared primary cultured WT and KO mouse embryonic fibroblasts (MEFs) and examined whether they exhibit an intrinsic difference in energy production. Indeed, KO MEFs possess a significant increase in ATP production compared to WT (Fig. 3b). The Seahorse XF Cell Mito Stress Test was conducted to assess mitochondrial respiration differences between cultured KO and WT MEFs, as such differences could affect ATP production. Following sequential treatments of oligomycin, carbonyl cyanide 4-(trifluoromethoxy) phenylhydrazone (FCCP), rotenone (Rot), and antimycin A, the oxygen consumption rate (OCR) was measured at four parameters of mitochondrial function: basal respiration, ATP turnover, proton leak, and spare respiratory capacity (Fig. 3c). The parameter calculations from the above measurements demonstrated that basal respiration, ATP production, maximal respiration, spare respiratory capacity, and non-mitochondrial respiration, were all increased in KO MEFs, while H^+^ (proton) leak did not significantly differ between WT and KO MEFs (Fig. 3d). Together, these findings suggest that the loss of *Fkbp51* induces higher mitochondrial function in energy metabolism as measured by ATP production.

### Co-localization of Parkin with FKBP51 protein in mitochondria

**Fig. 4:**
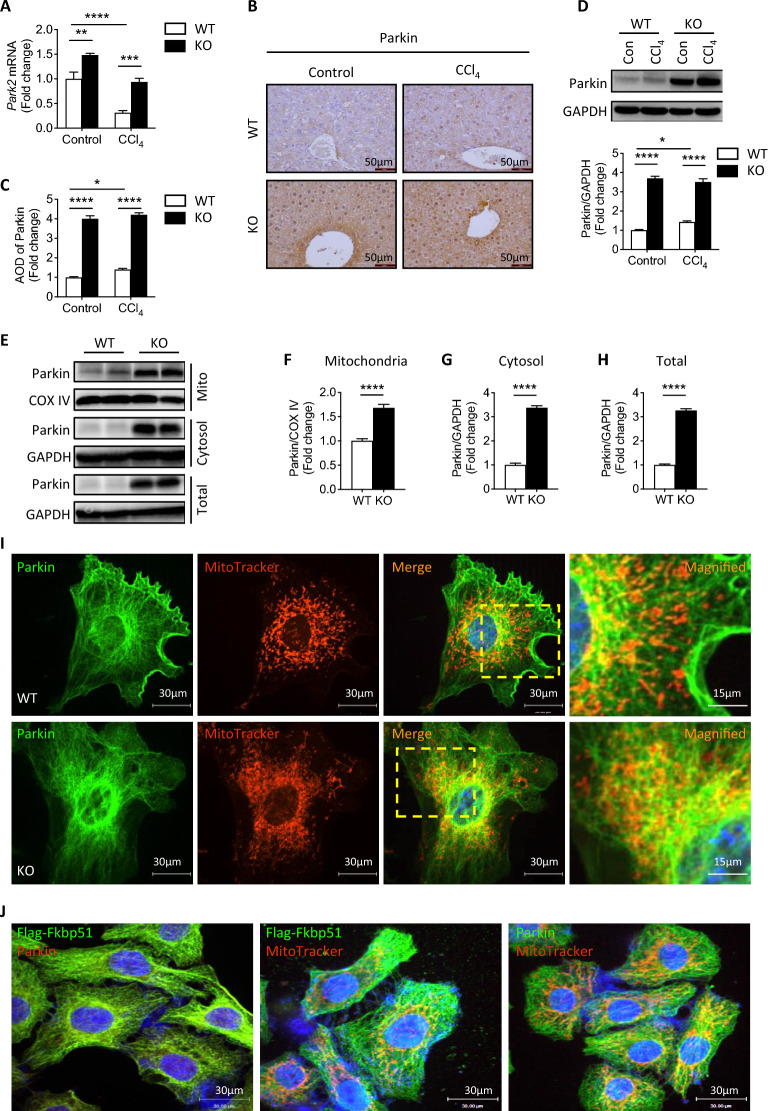
*Fkbp51* KO mice have a higher expression of Parkin than WT. **A** Quantitative PCR analyses reveal that *Fkbp51* KO liver expresses more *Park2* mRNA relative to WT in both control and CCl_4_-treated conditions. **B** Immunohistochemical labeling demonstrates an increase in Parkin in KO, and a CCl_4_–associated increase in both WT and KO. **C** Quantitation of AOD of Parkin in liver sections. **D** Western blotting confirms a higher level of Parkin in control (Con) KO liver, with an apparent increase of Parkin in WT following CCl_4_ treatment. **E**–**H** Increased Parkin expression in total KO liver lysates as well as in enriched mitochondrial and cytoplasmic fractions as compared to WT. Graphs represent mean values ± SEM from 6 mice for each group. *p* values were determined by student’s unpaired *t*-test (**F**–**H**) or two-way ANOVA (**A**, **C**, **D**) with the statistical significance labeled as follows: *as *p* < 0.05, **as *p* < 0.01, *** as *p* < 0.001 and **** as *p* < 0.0001. **I** A comparison of *Fkbp51* KO and WT MEFs confirms that the absence of FKBP51 is associated with higher levels of Parkin, more intense MitoTracker signal, and localization to the mitochondria. Magnified images outlined by the yellow boxes in the merged images are presented on right. Immunofluorescent labeling of Parkin and MitoTracker showed higher intensity of Parkin signal in KO than WT MEF cells, particularly in the perinuclear area and along the mitochondrial network. **J** Immunofluorescent labeling reveals the co-localization of FKBP51 and Parkin with mitochondria in HepG2 cells transfected with Flag-*Fkbp51*. Key: WT, wild type; KO, *Fkbp51* KO; AOD, average optical density; Con, control; Mito, mitochondria

Our above RNA-seq data indicate that *Fkbp51* KO possess upregulated *Park2* expression (Fig. 3a), a gene crucial for mitochondrial function and cytoskeleton formation [36–39]. We confirmed a higher *Park2* mRNA expression in KO mice compared to WT by real-time PCR (Fig. 4a). While CCl_4_ treatment decreased the mRNA expression in both groups, *Park2* was still higher in KO mice compared to WT (Fig. 4a). Based on these findings, Parkin protein expression in KO was studied using IHC and western blotting. Liver IHC revealed that Parkin expression is increased in KO mice when compared to WT, regardless of CCl_4_ treatment (Fig. 4b, c). Western blotting confirmed that Parkin protein levels are higher in KO mice versus WT both at baseline and following CCl_4_ treatment (Fig. 4d). Moreover, Parkin protein expression is higher in both the enriched mitochondrial fraction and cytosolic fraction in KO mice (Fig. 4e–h). We previously found that Parkin posttranslational modification (PTM) is regulated by FKBP51 protein in hippocampus, with no difference in *Park2* mRNA, but significantly higher Parkin expression in KO relative to WT [34]. Consistently, higher protein expression of Parkin was observed in KO livers as compared to WT under both control and CCl_4_ treatment. However, CCl_4_ treatment decreased the Parkin mRNA in WT and KO suggesting differences between tissue and treatment effects.

To understand these differences at the cellular level, mitochondria (MitoTracker labeling) and Parkin were tracked in WT and KO MEF cells. Parkin was expressed strongly in the cytoskeleton, more densely in KO MEFs than WT MEFs, particularly in the perinuclear area (Fig. 4i). A robust increase in mitochondrial network signal intensity of Parkin was observed in KO (Fig. 4i, magnified panel) and confirmed by Manders’ overlap coefficient score (Additional file 2: Fig. S4A), a result consistent with findings in western blotting (Fig. 4e, f). *Fkbp51* KO demonstrates a significant increase in Parkin expression (Fig. 4i and quantification in Additional file 2: Fig. S4B) and Parkin plays a critical role in mitochondrial function. This is in agreement with previously published co-immunoprecipitation (Co-IP) results demonstrating an interaction between Parkin and FKBP51 proteins [34]. Further identification of the co-localization of Parkin and FKBP51 could be an indication of their functional interaction within the cell. The HepG2 liver cell line was used for co-transfection experiments. Flag-*FKBP5*1 was transfected into HepG2 cells and was detected using an anti-Flag antibody. As shown in Fig. 4j, reduced expression of Parkin was observed due to higher FKBP51 expression, thus Flag-Fkbp51 and Parkin merged images show only weak Parkin labeling, a result consistent with our previously published mechanism. Overexpression of FKBP51 via Flag-Fkbp51 plasmid transfection led to a significant and dose-dependent decrease in endogenous Parkin expression (Fig. 4j) [34]. The Manders’ overlap coefficient score in Additional file 2: Fig. S4C demonstrates that FKBP51 is co-localized with Parkin. Additionally, in HepG2 cells, FKBP51 and Parkin were found to co-localize within mitochondria networks, with more intense labeling observed in the perinuclear area (Fig. 4j, middle and right, and Additional file 2: Fig. S4C). The co-localization of FKBP51 and Parkin in the mitochondria could partially explain how *Fkbp51* affects mitochondrial function, serving as a Parkin regulator.

### Ablation of FKBP51 increases mitochondrial size and induces autophagy/mitophagy in the liver

**Fig. 5:**
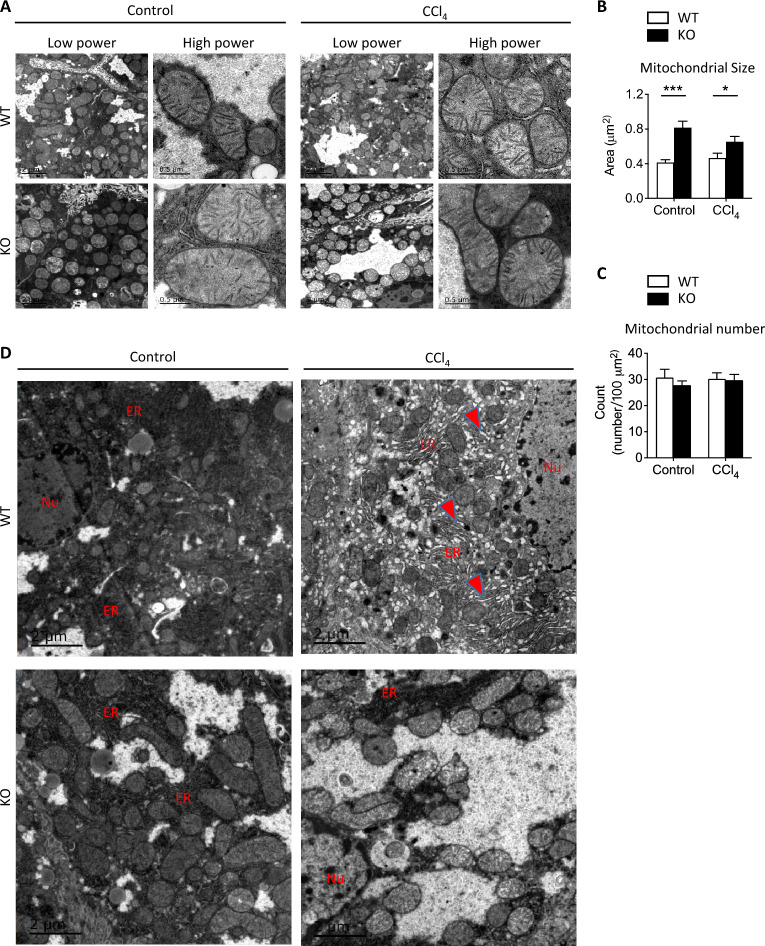
Liver mitochondria are larger in *Fkbp51* KO mice than in WT. **A** Representative EM photomicrographs of WT and KO liver mitochondria with or without CCl_4_ treatment. **B**, **C** Mitochondrial size (area) quantification demonstrated that *Fkbp51* KO mitochondria are larger in both control and CCl_4_ conditions, although no differences in mitochondrial number were detected. Quantifications are from random representative microphotographs measuring 250–300 mitochondria. *p* values were determined by two-way ANOVA with the statistical significance labeled as follows: *as *p* < 0.05, *** as *p* < 0.001. **D** Representative EM photomicrographs depict ER expansion in WT following CCl_4_ treatment. Key: WT, wild type; KO, *Fkbp51* KO; Nu, nucleus; ER, endoplasmic reticulum

To evaluate how the loss of FKBP51 affects mitochondrial function and Parkin-associated MQC at the subcellular level, mitochondrial morphology was assessed by electron microscopy (EM) [40]. Interestingly, KO liver cells were initially observed to possess larger mitochondria compared to WT mouse livers both at baseline and after CCl_4_ treatment (Fig. 5a). The quantification of randomly selected microphotographs, measuring 250–300 mitochondria, demonstrated that the KO exhibited significantly larger mitochondria than the WT, regardless of whether they were under control conditions or CCl_4_ treatment (Fig. 5b). Additionally, it was observed that CCl_4_ treatment had no impact on the size of mitochondria for either genotype, as compared to the control treatment (Fig. 5b). However, no difference in mitochondria number was observed between KO and WT in either condition (Fig. 5c).

**Fig. 6:**
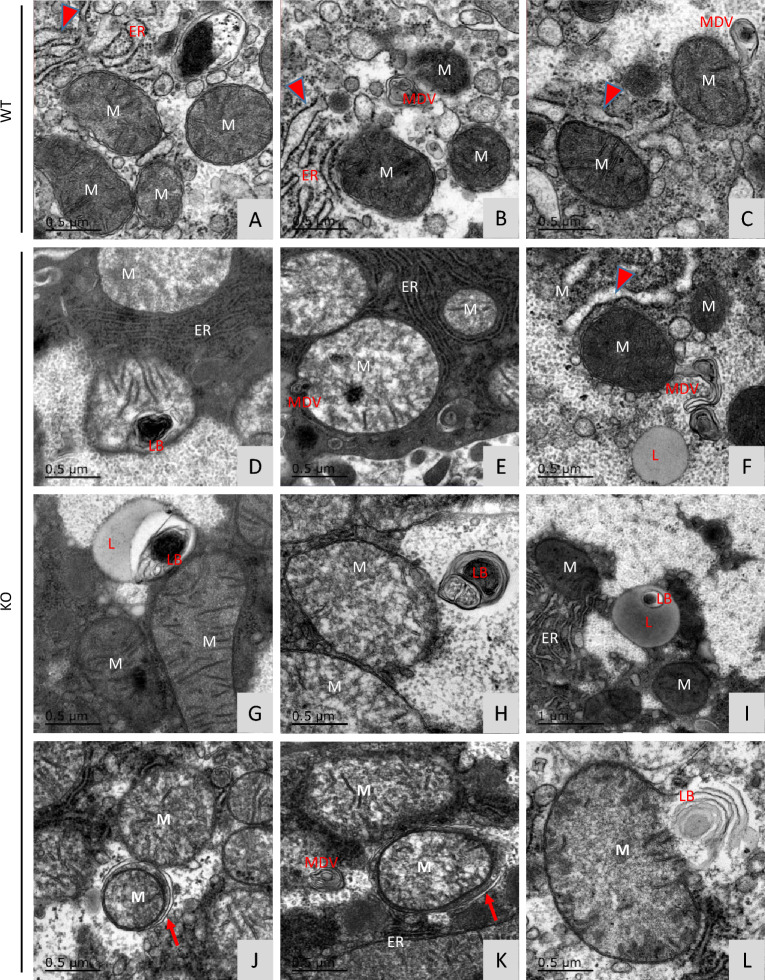
Representative EM images reveal more MDVs and mitophagy in *Fkbp51* KO liver after CCl_4_ injury. **A** ER expansion (red arrow) and **B** damaged mitochondria with incomplete membranes were observed more often in WT. Mitochondrial derived vesicles (MDV) were found in both WT (**B**, **C**) and KO (**E**, **F**, **K**), but more occurrence was found in KO. **D** Normal ER in KO with laminated bodies (LB) formed inside mitochondria. **E** Normal ER and dense particles observed inside mitochondria with multiple MDVs inside and at border of mitochondria. **F** Lysosome close to MDVs, and ER expansion (arrow) was occasionally observed around damaged mitochondria in KO. **G** Lysosome with a dense laminated body. **H** Laminated bodies with damaged granule bodies. **I** LB within lysosome with. **J**, **K** More mitophagy was observed in KO and representative mitophagosomes are indicated by the red arrows. **L** Mitochondria, ER, and LB in close contact during clearance of mitochondrial debris. Key: WT, wild type; KO, *Fkbp51* KO; ER, endoplasmic reticulum; M, mitochondria; MDV, mitochondrial derived vesicles; LB, laminated bodies; L, lysosome

No obvious ER morphological difference was observed between KO and WT in control treatment (Fig. 5d). After CCl_4_ treatment however, we observed more mitochondrial damage and ER expansion (arrowhead), with wrinkled and broken mitochondrial membranes (Fig. 6a–c), in WT mice, while the ER appeared normal in KO (Figs. 5d KO panel, 6d, e) with occasional ER expansion (Fig. 6f). The formation of mitochondrial-derived vesicles (MDVs) serves as a defense mechanism to remove harmful mitochondrial components and as a mechanism of immune tolerance and immune response. MDVs were observed in both but more often in KO compared to WT mice after CCl_4_ treatment (Fig. 6c in WT vs. Figure 6e, f, k in KO). Laminated bodies (LB) and MDVs to be released to the lysosome (L) were identified more prominently in KO mice (Fig. 6d, f, g, h, i, l). Remarkably, more occurrences of mitophagy (Fig. 6j, k, red arrows) were observed in KO mice versus WT after CCl_4_ treatment. The lighter appearance of some KO mitochondria was observed following CCl_4_ treatment, which may relate to its unique adaptation to insult (Fig. 5d and Additional file 2: Fig. S5). These EM images provided morphological evidence that KO mitochondria were better protected from CCl_4_ injury in than WT. To further identify molecular evidence to support these observations, protein-related to mitochondrial damage clearance (mitophagy) and MDV processes were studied.

**Fig. 7:**
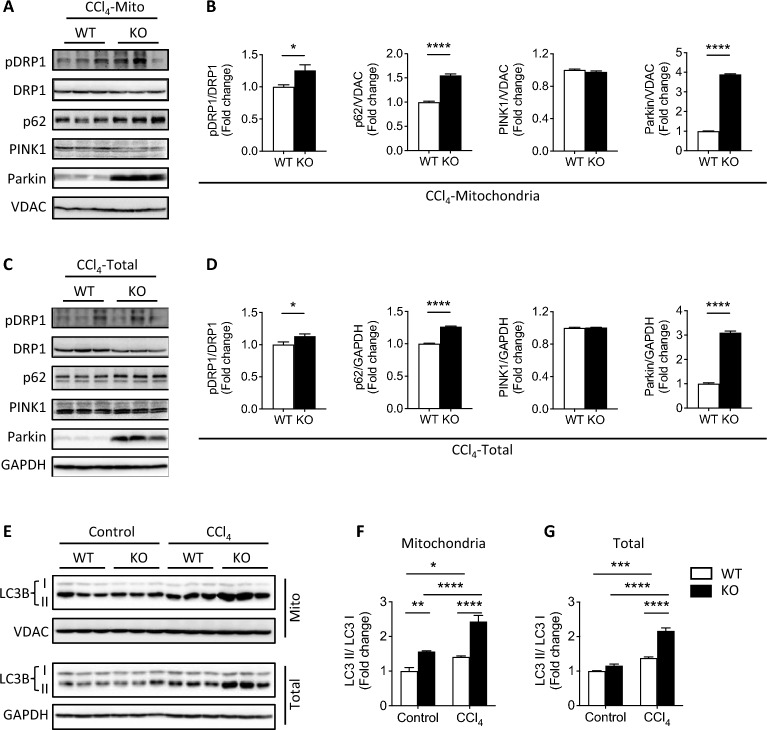
Increased autophagy/mitophagy in *Fkbp51* KO livers. **A**, **C** Immunoblots depict mitochondrial and total p-DRP1, DRP1, p62, PINK1, Parkin, and respective loading controls in CCl_4_-treated liver. **B**, **D** Densitometry ratios of target proteins relative to loading control in the mitochondrial and total fractions from **A** and **C**, respectively. **E**–**G** Immunoblots depict mitochondrial and total LC3B and respective loading controls in livers. Densitometry revealed higher LC3B II/I ratios in KO of mitochondria fraction and total protein following CCl_4_ treatment. Graphs represent mean values ± SEM from 6 mice for each group. *p* values were determined by student’s unpaired *t*-test for (**B**, **D**) or two-way ANOVA for (**F**, **G**) with the statistical significance labeled as follows: *as *p* < 0.05, **as *p* < 0.01, *** as *p* < 0.001 and **** as *p* < 0.0001. Key: WT, wild type; KO, *Fkbp51* KO; Mito, mitochondria

Mitophagy is an autophagy process for the removal of damaged mitochondria and is regulated by several important proteins including Parkin, dynamin-related protein 1 (DRP1), ubiquitin-binding protein p62 (also known as Sequestosome 1, an autophagy receptor), and PINK1 [41–43]. Consistent with results shown above (Fig. 4f–h), a significant increase of Parkin expression was found in the mitochondrial and total protein fractions of KO mice relative to WT after CCl_4_ injury. Relative to WT, KO exhibited higher p-DRP1/DRP1 ratios in both mitochondrial and total protein fractions, as well as increased p62 expression, but no change in PINK1 expression after CCl_4_ injury (Fig. 7a–d). Initialization of the mitophagosome requires microtubule-associated protein light chain 3B (LC3B) to degrade damaged mitochondria [44]. LC3B protein was measured in livers from untreated and CCl_4_-treated WT and KO mice. The ratio of LC3B II/I significantly increased after CCl_4_ treatment, particularly in KO mice, indicating increased autophagosome activity (Fig. 7e–g). The in vivo and in vitro data support a critical role for FKBP51 affecting mitochondrial dynamics, including increased occurrences of mitophagy and MDVs and less ER swelling in KO mice. Enhanced autophagy/mitophagy may explain the amelioration of CCl_4_-induced liver injury in the KO liver [40].

### *Fkbp51* KO MEF cells demonstrate increased autophagy/mitophagy marker expression and reduced ROS after CCCP treatment

**Fig. 8:**
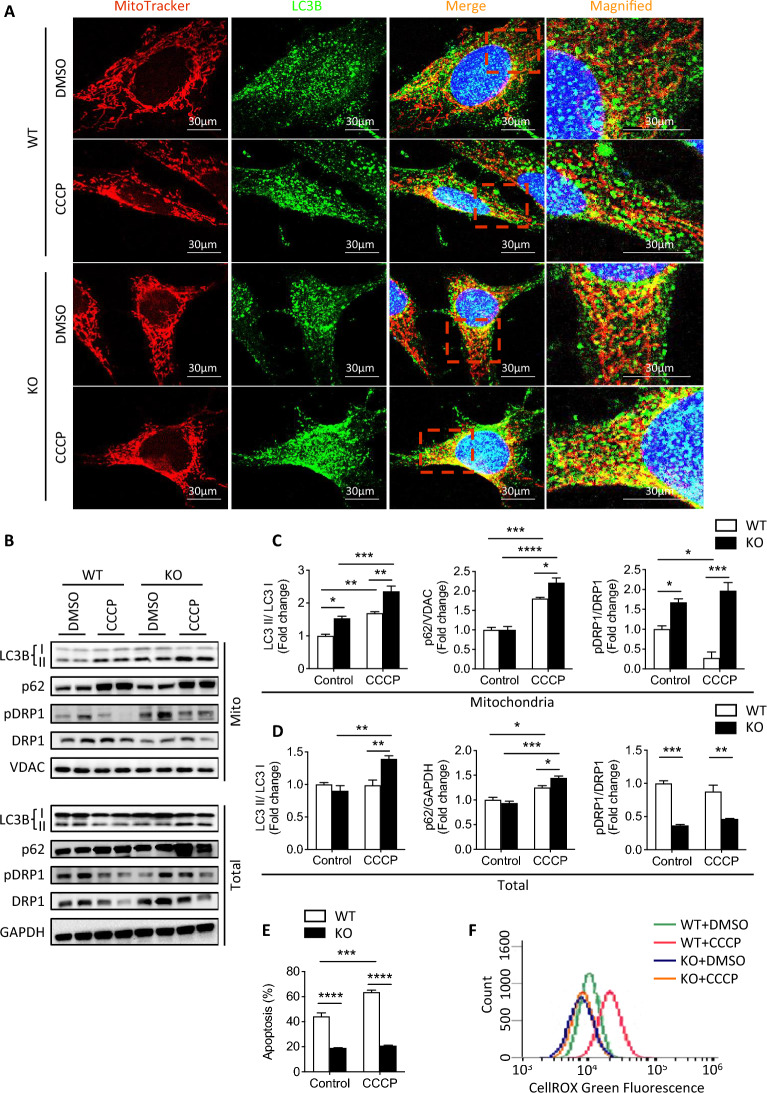
Treatment with CCCP enhances autophagy/mitophagy, particularly in *Fkbp51* KO. **A** *Fkbp51* KO and WT MEFs treated with DMSO or CCCP were assessed for the expression of the autophagy/mitophagy marker LC3. Both *Fkbp51* KO and WT exhibit increased expression of LC3, localizing to the mitochondria, with *Fkbp51* KO expressing the highest in both conditions. **B**–**D** Western blotting revealed KO exhibiting higher levels of LC3B II/I and p62/VDAC in mitochondrial and total fractions following CCCP treatment. Levels of pDRP1/DRP1 were higher in KO mitochondria, but lower in the total fraction. **E** Evaluation of apoptosis in control and CCCP treatment. **F** ROS levels were assessed by CellROX Green fluorescence intensity via flow cytometry. CCCP treatment showed a peak shift in WT MEFs only. Graphs represent mean values ± SEM from 3 independent experiments. *p* values were determined by two-way ANOVA with the statistical significance labeled as follows: *as *p* < 0.05, **as *p* < 0.01, *** as *p* < 0.001 and **** as *p* < 0.0001. Key: WT, wild type; KO, *Fkbp51* KO; Mito, mitochondria

To directly test whether FKBP51 plays a role in mitophagy, proton ionophore carbonyl cyanide m-chlorophenylhydrazone (CCCP), a mitochondrial uncoupler, was used to induce mitochondrial damage in primary cultured MEFs, and LC3B and p62, pDRP1, DRP1 were measured to assess mitochondrial stress [45]. Consistent with previous results, KO cells showed enhanced MitoTracker signal compared to WT, with decreased labeling intensity under CCCP treatment (Fig. 8a and quantification in Additional file 2: Fig. S6A). LC3B signals of both WT and KO MEFs were enhanced following CCCP treatment, with greater labeling intensity observed in KO MEFs (Fig. 8a and quantification in Additional file 2: Fig. S6B). Merged images of MitoTracker and LC3B indicate greater mitophagy in KO MEFs, as signified by a stronger yellow signal (Fig. 8a) and Manders’ overlap coefficient score (Additional file 2: Fig. S6C). Western blotting data support that the ratio of LC3B II/I is significantly higher in KO MEFs compared to WT after DMSO control treatment in the mitochondrial fraction and after CCCP treatment in both the mitochondrial and total protein fractions (Fig. 8b–d). Following CCCP treatment, p62 was increased in both fractions of KO and WT MEFs, though it was significantly higher in KO than in WT (Fig. 8b–d). Additionally, the mitochondrial p-DRP1/DRP1 ratio was higher in KO MEFs than WT before and after CCCP treatment but the total protein p-DRP1/DRP1 ratio was lower in KO than WT, suggesting that KO MEFs are more protected from CCCP-induced shifts in mitochondrial dynamics (Fig. 8b–d). The above data support an enhancement of autophagy/mitophagy function in *Fkbp51* KO MEF cells, protective mechanisms for preventing mitochondrial damage.

Although we demonstrated that more apoptotic cells are present in CCl_4_-treated WT liver than in KO (Fig. 1i, j), a direct difference of apoptosis was assessed resulting from mitochondrial damage induced by CCCP. In addition to confirming the presence of fewer apoptotic cells in KO, a significant increase of apoptosis was found in WT cells after CCCP treatment (Fig. 8e). Additionally, a CCCP-induced increase in the level of ROS was detected solely in primary cultured WT MEFs as indicated by peak shifting (Fig. 8f). This provided indirect evidence that KO cells produce fewer ROS during mitochondrial insult, which could partially explain the protective effect observed in KO mice following CCl_4_ treatment. Thus, these data support better MQC in *Fkbp51* KO through the regulation of mitophagy/autophagy processes, mitochondrial morphology and dynamics, and ATP production.

### Inhibition of FKBP51 with SAFit2 increases Parkin and ameliorates CCl_4_-induced liver injury

**Fig. 9:**
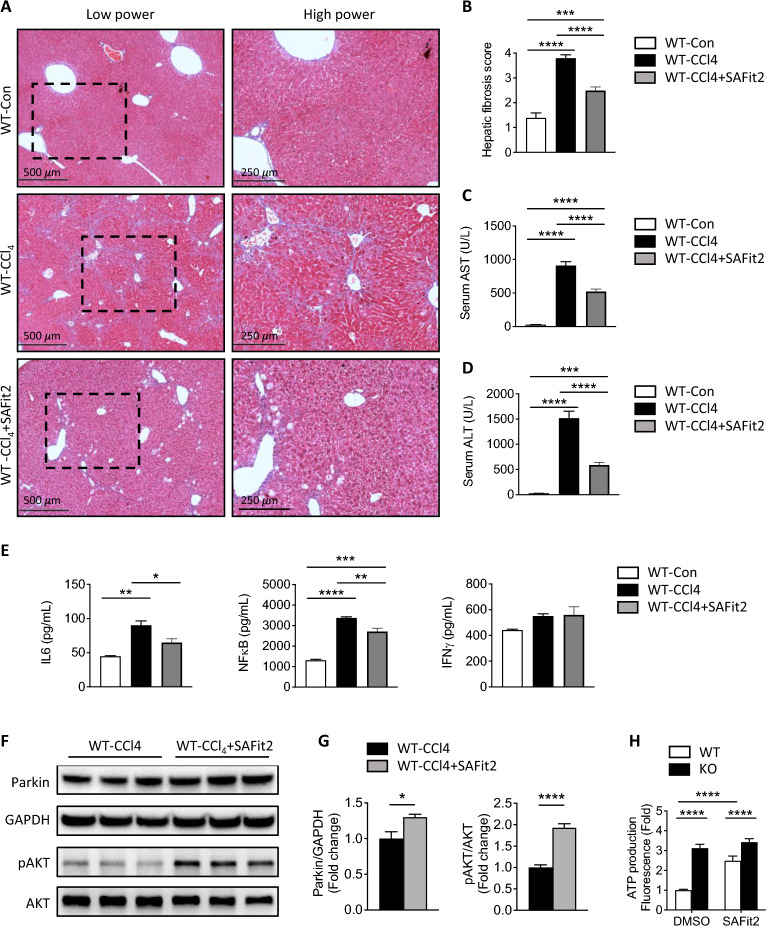
FKBP51 inhibitor SAFit2 attenuates CCl_4_-induced liver fibrosis and injury. **A** Masson’s trichrome staining of livers after treatment with SAFit2 in CCl_4_-induced liver fibrosis. **B** Assessment of liver fibrosis score. **C**, **D** AST and ALT levels are reduced with SAFit2. **E** SAFit2 application reduced serum IL6 and NFκB, but no effect on IFN-γ level. **F**, **G** Western blotting revealed an increase in Parkin and pAKT expression in WT-CCl_4_ treated with SAFit2 mice. Graphs represent mean values ± SEM from 5–6 mice for each group. **H** Normalized ATP production from WT and KO MEF cells treated with SAFit2. Graph represents mean values ± SEM from 3 independent experiments. *p* values were determined by student’s unpaired *t*-test (**F**), one-way ANOVA (**B**–**E**) or two-way ANOVA (**H**) with the statistical significance labeled as follows: *as *p* < 0.05, **as *p* < 0.01, *** as *p* < 0.001 and **** as *p* < 0.0001. Key: WT, wild type; KO, *Fkbp51* KO

We applied a highly specific selective FKBP51 inhibitor (SAFit2) to test its efficacy in preventing liver injury. SAFit2 has previously been applied in other studies and no toxicity was demonstrated with long-term treatment [46–48]. Concurrent injection of CCl_4_ and SAFit2 reduced indicators of liver injury, including lower intensity of trichrome staining (Fig. 9a) and lower quantified hepatic fibrosis scores (Fig. 9b). Serum AST and ALT levels were reduced in CCl_4_ + SAFit2-treated WT mice (Fig. 9c, d). Additionally, inhibition of SAFit2 led to a decrease in the levels of IL-6 and NFκB in the serum (Fig. 9e), which is in line with the observation that KO mice exhibit lower levels of IL-6 and NFκB (Additional file 2: Fig. S2). However, the inhibition by SAFit2 shows a lesser effect on IFN-γ (Fig. 9e). Consistent with the results from *Fkbp51* KO, SAFit2 inhibition was associated with a commensurate increase in Parkin (Fig. 9f, g). FKBP51 is a chaperone to AKT-specific phosphatase. Previously, elevated AKT phosphorylation was observed in Fkbp51 KO cells and SAFit2 inhibition of FKBP51 increased pAKT2 [11, 14]. To confirm the SAFit2 inhibition effect, pAKT and AKT were measured after SAFit2 application, and the increased ratio of pAKT/AKT suggested a consistent functional regulatory outcome (Fig. 9f, g). We further studied mitochondrial functional alterations after FKBP51 inhibition by measuring ATP production in vitro. WT and KO MEFs were treated with SAFit2, and ATP production was increased specifically in WT MEFs (Fig. 9h). The data support that inhibition of FKBP51 by SAFit2 prevents CCl_4_-induced liver injury, potentially via regulation of ATP production in the mitochondria.

## Discussion

In this study, we demonstrated that loss of FKBP51 function ameliorates liver injury partially via enhanced mitochondrial quality control. *Fkbp51* KO mice exhibited enhanced mitochondrial function (ATP production) and better protection of cellular function through mitochondrial protection (MDVs and mitophagy) after CCl_4_ injury. RNA-seq analysis pointed out multiple pathways and genes associated with this protective effect. Mitochondrial function-associated genes were enriched and we found that Parkin is significantly upregulated in KO in both basal and CCl_4_-treated conditions in addition to other genes with high-fold change and significant *p*-value. STRING analysis identified inter-connections between *Fkbp51*, *Hsp90aa1*, and *Park2*, and a hub of genes related to lipid and TAG biosynthesis and metabolism, fibrogenesis, immune response, cell cycle, and drug metabolism. We have previously demonstrated the interaction of FKBP51 and Parkin using immunofluorescence (IF) and co-immunoprecipitation [34]. In the current study, we found that these two proteins co-localize in mitochondria. Treatment with the selective FKBP51 inhibitor SAFit2 also substantiated its effectiveness as a means of reducing liver injury, confirming FKBP51 to be a useful therapeutic target. Together, these findings support the conclusion that targeting FKBP51 represents a novel mechanism to protect against liver injury.

Previously, we demonstrated the prevention of HFD-induced fatty liver and alcohol-induced liver injury using our *Fkbp51* KO model [13, 18]. In our current study, the first line of evidence includes the results from the histological and biochemical analyses and the observed downregulation of multiple genes that are functionally related to fibrogenesis in KO. For example, downregulation of the α-2 macroglobulin (*A2m*) gene and TIMP1 protein were found in *Fkbp51* KO following CCl_4_ treatment. In human research, the serum levels of these two markers are reliable predictors of NASH and alcoholic liver disease risks [50]. Secondly, genes involved in increasing TAG degradation and reducing TAG biosynthesis were found in *Fkbp51* KO mice. The downregulation of Patatin-like phospholipase domain-containing protein 3 (*Pnpla3*) in KO mice is consistent with human research, where genetic variants of *PNPLA3* have been linked to non-alcoholic steatohepatitis (NASH) and NAFLD [51], and higher liver TAG content [52]**.** Increased levels of the NAFLD-linked PNPLA3 isoform result in larger lipid droplets, while decreasing PNPLA3 results in an opposite trend [53]. Thus, lower expression of *Pnpla3* in *Fkbp51* KO suggests the potential for less lipid deposition, consistent with reduced TAG biosynthesis and enhanced lipid metabolism. Previous studies using our *Fkbp51* KO highlight the importance of FKBP51 in GR and PPARγ function, potentially through the regulation of the AKT and p38 pathways [13, 14]. In WT mice, activation of PPARγ results in elevated PPARγ-induced genes, such as lipoprotein lipase (LPL), which is moderately lower in *Fkbp51* KO [13–15]. In our study, we found the PPAR signaling pathway to be significantly altered in CCl_4_-treated KO and WT (Table 1). Upstream regulator effects analysis predicted PPARγ activation in KO after CCl_4_ liver injury (Fig. 2c). However, the activation of PPARγ resulted in upregulation of LPL to a much higher degree in WT (8x) than KO (2x). Activation of GR and PPARγ in this model needs to be investigated further. Interestingly, the expression of genes involved in fatty acid biosynthesis were downregulated in *Fkbp51* KO after CCl_4_ injury, including *Lpin1*, *Acsl3*, and *Acsl4*. Mice lacking *Lpin1* demonstrate faster recovery from endotoxin administration and enhanced autophagic clearance; results consistent with the protection from liver injury observed in *Fkbp51* KO mice [54]. Additionally, we found increased expression of CYP450 member genes including Cyp2A5 and Cyp2c39. Cyp2A5 protects against ALD development and drug-induced liver injury [55, 56], and upregulation of Cyp2c39 expression may reduce liver retinoic acid accumulation and liver fibrosis due to its function in retinoic acid catabolism [57]. Pathway and function analysis pointed out that inflammation, mitochondria, and oxidative metabolism are physiological systems likely involved in the protection of liver injury in *Fkbp51* KO mice. Thus, multiple lines of evidence from gene expression, biomedical function, and physiological alterations in the *Fkbp51* KO are consistent with human findings.

FKBP51 was first identified as a GR binding protein, negatively regulating GR activity, but further studies revealed its role in post-translational protein modification (PTM) [5, 58]. Consistently, *Fkpb51* elimination enhances GR inhibition and lowers glucocorticoid expression in serum. Previous research found that FKBP51 directly regulates AKT phosphorylation and participates in autophagy processes [59] and other protein PTM [60–62]. In our study, FKBP51 inhibition by SAFit2 results in up-regulation of pAKT, which is consistent with previous observations of their relationship. Recently, we have found that FKBP51 directly binds to Parkin and affects its PTM [34]. Our data suggests the potential involvement of FKBP51 in the autophagy/mitophagy process via upregulation of Parkin, as Parkin is known to play a critical role in mitophagy [63, 64]. As an important regulator of mitophagy and mitochondrial function, Parkin protects against alcohol-induced liver injury and steatosis [39]. Additional studies of Parkin-associated mitochondrial proteins determined that acyl-CoA synthetase long-chain family members ACSL1 and ACSL4 are involved in mitochondrial metabolism [65]. Interestingly, we found that *Acsl3* and *Acsl4* are downregulated in *Fkbp51* KO and that they share a gene hub with *Park2*. Parkin is a known critical member of MQC processes, including mitophagy and MDVs, and we propose that FKBP51 participates in these processes through the regulation of Parkin activity, as evidenced by the increased number of MDVs observed in KO. Thus, regulation of Parkin and mitochondrial quality control by FKBP51 represents a promising avenue for disease treatment, and could also enhance related functions including the degradation of misfolded proteins, autophagy/mitophagy, and MDVs [66, 67].

FKBP51 is a co-chaperone of HSP90 and functions as an isomerase, which is critical for protein folding and binding. This data set also suggested protein binding activity to be the most significant altered molecular function. By inhibiting mitochondria-localized members of the HSP90 family, cytosolic HSP90 was shown to be required for the proper folding of PINK1 [68]. Mitochondrial HSP90 is needed for protein import and the assembly of a multiprotein complex activating PINK1- and Parkin-dependent MQC. It is also possible that FKBP51/HSP90 plays a cochaperone function in the folding and disaggregation of proteins in mitochondrial compartments and participates in this MQC. Consistently, *Hsp90aa1* gene expression is found to be downregulated in *Fkbp51* KO following liver injury. RNA-seq data suggests that mitochondrial function associated genes are enriched, and the noted increase in ATP production and reduction in ROS production after liver injury indicate enhanced mitochondrial function. Increased mitophagy was evidenced by EM and molecular markers. Thus, multiple lines of evidence suggest that *Fkbp51* is a critical gene for mitochondrial function and quality control via regulation of one or more key components.

CCl4-induced liver injury and fibrosis have resulted in immune response and microphages infiltration (20, 21). In our study, GO analysis predicted the functional including decreased inflammatory response and increased lipid metabolism (Table 3). As an immunophilin protein, FKBP51 functions in immune response and mitochondrial function. It has been demonstrated that FKBP51 shuttling between the mitochondria and the nucleus is involved in the regulation of proinflammatory cytokines, and that its depletion reduces the expression of type I IFN following infection [20]. Furthermore, FKBP51 was demonstrated to play roles in the control of NFκB and TGFβ signaling, whereas the inhibitory immune checkpoint programmed cell death 1 (PD1) and its ligand (PD-L1) promote alternative splicing of the *FKBP51* gene [69]. The silencing of FKBP51 results in reduced cytokine and chemokine secretion [70]. It is likely that the protection conferred to KO mice may be due in part to the minimal increases in some immune factors including TGFβ, NFκB, IL6, and many more after CCl_4_ treatment in our study. Pathway analysis in our research has identified that the top regulatory differences in the WT and KO response to CCl_4_ injury are the engulfment of myeloid and endocytosis. Myeloid cells play a major role in the sensitization to liver injury, and the myeloid cell-liver axis is critically important in chronic liver disease [71]. Thus, these results have begun to illuminate immune-specific roles of *Fkbp51* in CCl_4_-induced liver injury. More specifically, our findings are in line with prior research on these immune function-related genes. The KO mice also downregulated immune-relevant genes, such as lipocalin 2 (*Lcn2*), which is critical in innate immunity. Previous studies found that *Lcn2* KO mice have enhanced mitochondrial function and differential redistribution of linoleic acid in the inner mitochondrial membrane [72], and *Lcn2* is critical for cell proliferation, autophagy, and mitochondrial biogenesis and liver function [73, 74]. Other immune function-related genes are also worth mention here, such as CD14, Il1r1, and Tiam2 are downregulated in KO after CCl_4_ injury. CD14 is a surface antigen and mediates innate immune response, CD14 controls Toll-like 4 endocytosis and is implicated in liver disease [75–78]. T-lymphoma invasion and metastasis-inducing protein 2 (Tiam2) is required for signal transduction pathways involved in the regulation of cytokinesis and implicated in liver physiology and cancer development were found down regulated in *Fkbp51* KO [79, 80]. The function of FKBP51 in regulating the immune response to liver injury remains a question warranting further investigation.

One limitation of this research is that the in vitro molecular analyses use MEF cells and hepatocytes. In the future, genetic modification of *Fkpb51* in specific liver cell types should be considered to understand the role of *Fkbp51* in fibrosis. Macrophages and Kupffer cells should be considered first due to their high expression of *FKBP51* in humans (https://www.proteinatlas.org/ENSG00000096060-FKBP5). It is important to note that the induction levels of IL-6 and NFκB in response to CCl_4_ treatment in WT mice (Fig. 9e) appear to be inconsistent with the results depicted in Additional file 2: Fig. S2, which show no significant change in WT mice with and without CCl_4_. We have identified two notable factors that may contribute to these variations. Firstly, there is a difference in the injection schedule (Additional file 2: Fig. S7), and secondly, DMSO was used as a solvent control in Fig. 9e (further details in the Additional file 2: Fig. S7 and method section). It is possible that the results can be explained by the fact that DMSO can modulate the activity of NKT and NK cells [49].

In summary, our study highlighted the impact of *Fkbp51* in multiple pathways and genes that are associated with better endo-phenotypes (liver enzymes, histology, pro-inflammatory factors) in *Fkbp51* KO mice after CCl_4_-induced liver injury. Knockout of *Fkbp51* protects against CCl_4_-induced liver injury via the promotion of MQC (including the enhancement of mitochondrial respiration, reduced oxidative stress, and apoptosis), downregulation of some immune response-related genes, increased expression of some Cyp450 family members, and increased mitophagy. Additionally, the downregulation of cell cycle-associated genes after liver injury in *Fkbp51* KO echoes its function in cell proliferation and cancer [81–84]. FKBP51 regulation of Parkin activity in the liver is a novel mechanism to explore for future treatments making the inhibition of FKBP51 a promising strategy in the treatment of liver injury.

## Materials and methods

### Animals

All experimental protocols were approved by the Animal Care and Research Advisory Committee of the Institute of Laboratory Animal Science, Chinese Academy of Medical Sciences & Peking Union Medical College, Beijing. The animals were maintained in facilities fully accredited by the Association for the Assessment and Accreditation of Laboratory Animal Care (AAALAC). Development of *Fkbp51* KO mice was described in a previous publication [85]. *Fkbp51* KO and WT littermates were bred through heterozygous (*Fkbp51* ±) mating. Only male mice were used in this study. WT mice weigh approximately 25 g at 8 weeks of age, while KO weigh approximately 23 g.

### Mouse model of liver fibrosis

Male 8-week-old WT and *Fkbp51* KO mice were randomly assigned into either olive oil injection control or CCl_4_ injection treatment groups. Modified protocol was followed from previous publication [35, 86]; specifically, mice were injected intraperitoneally (IP) three times weekly for 2 weeks with olive oil (vehicle, sigma-aldrich, MO, USA) or carbon tetrachloride (CCl_4_, sigma-aldrich, MO, USA) diluted 1:9 in olive oil at a dose of 5 μl/g of body weight (BW). A schematic diagram illustrating the injection procedure can be found in Additional file 2: Fig. S7a. Mice were sacrificed 48 h after the final injection, and their livers were harvested after being perfused with ice-cold saline, except in the case of Fig. 1a. For Fig. 1a, the purpose was to compare and analyze the overall liver phenotype among the groups, including evaluating the original color displayed by each group.

### Histological analysis and measurements of serum cytokines

Paraffin embedded liver sections at 4 μm thickness were prepared for Masson's trichrome, immunohistochemistry (IHC), and TUNEL labeling. Masson’s trichrome staining was performed following the manufacturer’s instructions (Abcam, MA, USA) and photomicrographs were acquired using a CTR6000 microscope with a DFC450 C camera (Leica, Wetzlar, Germany). The hepatic fibrosis score was evaluated from eight randomly selected fields, as described [32]. IHC was performed as previously described [34]. A list of antibodies used in this study is included in Additional file 1: Table S3. DAB-based TUNEL assay kit (#ab206386, Abcam, MA, USA) was used to measure apoptosis in the liver according to the manufacturer’s instructions. After mice were anesthetized by 0.02 g/ml tribromoethanol (18 µl/g BW), blood was collected from the abdominal aorta using coagulation tubes (BD, NJ, USA). Supernatants were collected for the measurement of cytokines with ELISA detection kits according to the manufacturer’s instructions. A list of ELISA kits used in this study is included in Additional file 1: Table S4.

### RNA-seq data analysis

RNA-seq expression profiling was performed on vehicle control- and CCl_4_-treated KO (n = 3) and WT (n = 3) mice (n = 12 total). Liver tissues were snap frozen and RNA was isolated using TRIzol^*®*^ followed by RNeasy Mini kit purification (Qiagen, Hilden, Germany). Sequencing and data analysis were performed as previously published [87], with the exception that the reads were aligned to the reference *Mus musculus* genome (UCSC build MM9) with TopHat [88]. DEGs were identified between KO and WT at different *p*-value cut offs. Multiple test adjustments were also performed to control for false positives with an adjusted (adj) *p*-value of < 0.05 using the Benjamini–Hochberg adjustment [89] and sequence read abundances with readCount > 50 or rpkm > 1. The significant genes with Fold Change (FC) > 2 were analyzed further. Heatmaps of gene expression levels were generated with R/heatmap2. Genes that were differentially expressed between KO and WT following treatment were analyzed using IPA, Ingenuity^®^, and GO enrichment analysis.

### Cellular ATP, metabolism assays, and immunocytochemistry (ICC)

Primary WT and *Fkbp51* KO MEFs were cultured and passaged as previously described [85]. Cellular ATP synthesis in WT and KO MEFs was determined by PhosphoWork Luminometric ATP Assay Kit (AAT Bioquest, Inc, CA, USA) according to the manufacturer’s protocol. The cells were lysed in 100 μl ATP assay solution and the luminescence intensity was recorded on a luminometer (Thermo Fisher Scientific, MA, USA). ATP production was normalized to protein concentration measured by the BCA method. Mitochondrial function was evaluated by XF Cell Mito Stress Test using the XF24 Extracellular Flux Analyzer (Seahorse Bioscience, MA, USA) according to the manufacturer’s instruction. Briefly, cells were seeded in the XF24 cell culture microplate at 1 × 10^4^ cells/well the day before testing. Cells were washed and cultured in pre-warmed assay medium (XF Base Medium contained with 5 g/L glucose, 2 mM pyruvate, 2 mM L-glutamine, pH 7.4) and placed in an incubator without CO_2_ for 1 h prior to the assay. After measurement of baseline oxygen consumption rate (OCR), the hydrated sensor cartridges were loaded with oligomycin (1 μM), FCCP (1 μM), and rotenone (0.5 μM) to measure ATP-related OCR, maximal respiration-related OCR and non-mitochondrial respiration-related OCR, respectively. The data were calculated with the XF Stress Test Report Generator according to the user guide and normalized to protein concentration. To induce mitophagy, primary WT and KO MEFs were treated with CCCP (Sigma-Aldrich, MO, USA) at a final concentration of 10 μM for 18 h. HepG2 cells were cultured in Dulbecco's Modified Eagle Medium (DMEM, Gibco, NY, USA) containing 10% fetal bovine serum (Gibco) and penicillin (Gibco). The cells were transfected with Flag-*Fkbp51* (HG11487-CF, Sino Biological, Beijing, China) using lipofectamine 3000 (Thermo Fisher Scientific, MA, USA) according to the manufacturer’s recommendations. MitoTracker Red (Thermo Fisher Scientific, MA, USA) was used to identify mitochondria according to manufacturer’s instruction. Briefly, after indicated treatment, the cells were incubated with MitoTracker at a final concentration of 200 nM at 37ºC for 30 min. After being washed with warmed DMEM and PBS, the cells were fixed with 4% paraformaldehyde, and ICC was performed using anti-FKBP51, anti-Parkin, or anti-LC3 antibodies as previously described [34]. The fluorescence signal was captured using confocal laser scanning microscopy (Leica TCS LSI, Germany). A list of antibodies used in this study is included in Additional file 1: Table S3. The quantitative colocalization was performed using Manders' overlap coefficient determined by Fiji ImageJ software with the BIOP Jacop plugin [90]. This coefficient will vary from 0 to 1, the former corresponding to non-overlapping images and the latter reflecting 100% co-localization between both images. The relative normalized mean fluorescence intensity (MFI) of target protein per cell was quantified using ImageJ.

### Isolation of mitochondria from liver and MEF cells

Either 200 mg minced liver tissue or 10^6^ MEF cells were homogenized with 1 ml isolation separating medium (containing 5 mM HEPES, 220 mM mannitol, 70 mM sucrose, 1 mM PMSF, 0.2% BSA, and 1 μg/ml aprotinin, pH 7.4) as described [91]. After centrifuging at 1,000 × g for 10 min at 4 °C, the supernatants were collected and centrifuged at 10,000 × g for 10 min at 4 °C. The cytoplasmic fraction in supernatants were collected and the mitochondria in pellets were resuspended with 5 volumes of reserve medium (separating medium without BSA and aprotinin). The protein concentrations were determined using the Pierce BCA kit (Thermo Fisher Scientific, IL, USA).

### Western blotting and quantitative real-time PCR (qPCR)

Proteins from liver were harvested in lysis buffer with 1:10 volume of protease inhibitor and 1:100 volume of phosphatase inhibitor cocktail (Roche, IN, USA) as previously described. Western blotting was performed as previously described [92–94]. The primary antibodies used in this research include anti-COX IV, -DRP, -p-DRP, -FKBP51, -GAPDH, -LC3B, -P62, -Parkin, -Pink1, and -VDAC. Detailed information for these antibodies and vendors is listed in Additional file 1: Table S3. Signals were captured and quantified using the Chemiluminescent Imaging System. Liver mRNA was isolated using TRIzol^®^ (N = 3–5). Reverse transcription (RT) and qPCR were conducted according to the manufacturer’s instructions (TaKaRa Biotechnology, Dalian, China) using the ABI PRISM 7500 System (Thermo Fisher Scientific, MA, USA). The relative mRNA expression levels were normalized to *Gapdh*, which was not differentially expressed between KO and WT groups. Primer sequences are listed in Additional file 1: Table S5.

### Transmission electron microscopy (EM)

Mouse liver tissues were cut into approximately 1 mm cubes, fixed in 2.5% Glutaraldehyde in 0.1 M Phosphate Buffer (pH 7.4), and post-fixed in 1% osmium tetroxide. The samples were then rinsed with sodium cacodylate buffer and dehydrated with gradient alcohol, replaced by propylene oxide and embedded in Epon 812 (Sigma-Aldrich). Semi-thin Sects. (1 μm) were cut, stained by methylene blue, and oriented under a light microscope. Ultra-thin sections were stained with uranyl acetate and lead citrate and were monitored under a JEM-1400 electron microscope (JEOL, Tokyo, Japan). The mitochondrial size from around 200 fields for each group were measured by Fiji *Image J* software.

### Cellular apoptosis and reactive oxygen species (ROS) analysis

Primary WT and KO MEFs were cultured with or without CCCP (10 μM) for 18 h. Apoptotic cells were detected using 10 μl/ml Annexin V (BioVision, CA, USA). Data were acquired with a BD FACS Canto II flow cytometer and analyzed with FlowJo (Treestar, OR, USA). For ROS measurement, the cells were treated with CellROX Green (Thermo Fisher Scientific, MA, USA) at a final concentration of 5 μM and incubated at 37 °C for 30 min. After washing three times with warmed PBS, the cells were collected, and the ROS level was determined by flow cytometry (BD Biosciences, CA, USA). The fluorescence intensity was determined from 10,000 cells per sample. The data were analyzed using BD Csampler Software and displayed in Histogram (BD Biosciences, CA, USA).

### In vivo and in vitro SAFit2 treatment

Male WT mice were randomly assigned to one of three groups (i) Control injection, (ii) CCl_4_ injection, or (iii) SAFit2 concurrent with CCl_4_ injection (n = 5–6 mice/group). CCl_4_ was delivered at the same dosage as the earlier experiment three times a week for 2 weeks. The stock solution of SAFit2 (10 mM in 623 µl DMSO) was diluted in 0.9% NaCl to achieve a final volume of 12 ml, resulting in a DMSO concentration of 5% v/v. The SAFit2 was administered via intraperitoneal injection on the day following the CCl_4_ injection, with a dosage of 12 µl/g of body weight. The final dosage of SAFit2 was 6 mg/kg of body weight per day; a dose that has been used in other studies and is well tolerated over a prolonged period of time [11, 95]. As a control for SAFit2, an equal concentration of DMSO (5% v/v) dissolved in saline was injected. A schematic diagram illustrating the injection procedure can be found in Additional file 2: Fig. S7B. Liver and blood were collected for liver histology, protein assays, and serum AST and ALT measurement. MEFs from KO and WT were treated with SAFit2 at 30 µM for 12 h and ATP levels were measured.

### Statistical analysis

All values are presented as mean ± standard error of the mean (SEM). Comparisons between two groups were performed using Student’s *t*-test, while comparisons for multiple group differences were performed using one-way analysis of variance (ANOVA) or two-way ANOVA, followed by a Student-Newman–Keuls test. GraphPad Prism was used for data analysis (GraphPad Software Inc.), and significance was defined as *p* < 0.05.

### Supplementary Information

**Additional file 1. Table S1**: Gene list of IPA Pathway. **Table S2**: DEGs with high fold change. **Table S3**: Antibody list for Western blotting, IHC, and IF. **Table S4**: ELISA kit for serum cytokines analysis. **Table S5**: Primer list for qRT-PCR.

**Additional file 2. Fig. S1**: Immunohistochemical comparisons of Control and CCl4-treated *Fkbp51* KO and WT liver sections. Expression patterns of **A** Collagen I, **B** CTGF, and **C** α-SMA are all higher in the livers of CCl4 treated WT mice than in KO mice. Key: WT, wild type; KO, *Fkbp51* KO. **Fig. S2**: Serum analyses of Control and CCl4-treated *Fkbp51* KO and WT were performed to determine the concentrations of **A** IFN-γ, **B** IL-6, **C** TGF-β1, **D** NFκB, **E** IL-10, **F** TNF-α, **G** GC, and **H** FGF. Graphs represent mean values ± SEM from 6 mice for each group. *p* values were determined by two-way ANOVA with the statistical significance labeled as follows: *as *p* < 0.05, ** as *p* < 0.01 and **** as *p* < 0.0001. Key: WT, wild type; KO, *Fkbp51* KO. **Fig. S3**: **A** IPA pathway analysis identified disease and development, physiological system and development, signaling pathways, and toxicity-related changes as different between KO and WT after CCl4 injury. **B** Go analysis further suggested the enrichment of biological process, cellular component, and molecular function. The top 10 relevant pathways are included in the bar graph. **Fig. S4**: The quantification for Fig. 4i, j. **A** Increased cololization of Parkin/MitoTracker in KO MEFs. **B** The increased relative normalized MFI of Parkin per cell in KO MEFs. **C** Colocalization between Flag-FKBP51/Parkin, Parkin/Flag-FKBP51, Flag-FKBP51/MitoTracker and Parkin/MitoTracker. Graph represents mean values ± SEM from 3 independent experiments. *p* values were determined by student’s unpaired *t*-test (**A**, **B**) with the statistical significance labeled as follows: *** as *p* < 0.001, **** as *p* < 0.0001. Key: WT, wild type; KO, *Fkbp51* KO. **Fig. S5**: Representative EM photomicrographs demonstrate that KO has less ER expansion than WT, and KO liver mitochondria are lighter in appearance than WT mitochondria after CCl4 treatment. **Fig. S6**: The quantification for Fig. 8a. **A**, **B** The relative normalized MFI of Mitotracker and LC3B per cell from both genotype under DMSO or CCCP treatment. **C** Colocalization between LC3B/MitoTracker. Graph represents mean values ± SEM from 3 independent experiments. *p* values were determined by two-way ANOVA with the statistical significance labeled as follows: *** as *p* < 0.001, **** as *p* < 0.0001. Key: WT, wild type; KO, *Fkbp51* KO. **Fig. S7**: Schematic diagrams depicting the injection procedures for the animal models.

## Data Availability

The datasets generated during and/or analyzed during the current study are not publicly available due to an additional publication is in preparation using this data set but are available from the corresponding author on reasonable request.

## Abbreviations

ACOX1: Acyl-CoA oxidase 1
Acsl3: Acyl-CoA synthetase long chain family member 3
AHR: Aryl hydrocarbon receptor
AKT/PKB: AKR mouse strain that develops spontaneous thymic lymphomas/protein kinase B
ALD: Alcoholic liver disease
ALT: Alanine aminotransferase
AST: Aspartate transaminase
ATP: Adenosine triphosphate
CCCP: Carbonyl cyanide m-chlorophenylhydrazone
CCl_4_: Carbon tetrachloride
COXIV: Cytochrome C oxidase IV
CTGF: Connective tissue growth factor
DEG: Differentially expressed genes
DHT: Dihydrotestosterone
DRP1: Dynamin-related protein 1
EM: Electron photomicrographs
ER: Endoplasmic reticulum
Ethe1: Ethylmalonic encephalopathy protein 1
FCCP: Carbonyl cyanide 4-(trifluoromethoxy) phenylhydrazone
FGF: Fibroblast growth factor
FKBP51: FK506 binding protein 51
GC: Glucocorticoid
Gls2: Glutaminase 2
GR: Glucocorticoid receptor
HCC: Hepatocellular carcinoma
HPA axis: Hypothalamus–pituitary–adrenal axis
HSC: Hepatic stellate cell
HSP90: Heat-shot protein 90
ICC: Immunocytochemistry
IF: Immunofluorescence
IFN-γ: Interferon-γ
IHC: Immunohistochemistry
IL1: Interleukin-1
IP injection: Intraperitoneal injection
IPA: Ingenuity pathway analysis
KEGG: Kyoto encyclopedia of genes and genomes
KO: Knockout
LC3B: Microtubule-associated protein light chain 3B
*Lcn2*: Lipocalin-2
LEP: Leptin
*Lipin1*: Lipin 1
LPS: Lipopolysaccharides
LXR: Liver X receptor
MDVs: Mitochondrial-derived vesicles
MEF: Mouse embryonic fibroblast
MQC: Mitochondrial quality control
NAFLD: Non-alcoholic fatty liver disease
NASH: Non-alcoholic steatohepatitis
NFκB: Nuclear factor-κB
OCR: Oxygen consumption rate
p62/SQSTM1: Ubiquitin-binding protein P62/sequestosome 1
PD-1/PD-L1: Programmed cell death 1 and its ligand
PINK1: PTEN Induced Kinase 1
*Pnpla3*: Patatin-like phospholipase domain-containing protein 3
PPARγ: Peroxisome proliferator-activated receptor gamma
Prodh: Proline dehydrogenase
PTM: Post-translational protein modification
ROS: Reactive oxygen species
RXR: Retinoid X receptor
SAFit2: A selective inhibitor of FKBP51
SEM: Standard error of the mean
Slc8b1: Solute carrier family 8 member b1
STRING: Search Tool for the Retrieval of Interacting Genes
Suox: Sulfite oxidase
TAG: Triacylglyceride
TGF-β1: Transforming growth factor-β1
TIMP1: Tissue inhibitor of metalloproteinase 1
TNF-α: Tumor necrosis factor-α
Tomm40l: Translocase of outer mitochondrial membrane 40 like
TUNEL staining: Terminal deoxynucleotidyl transferase deoxyuridine triphosphate nick end staining
WT: Wildtype
α-SMA: Alpha smooth muscle actin
