# Supplementary material for: Knocking out Fkbp51 decreases CCl4-induced liver injury through enhancement of mitochondrial function and Parkin activity
Source: Cell Biosci. 2024 Jan 2;14:1. doi: 10.1186/s13578-023-01184-3 (PMC10763032; doi:10.1186/s13578-023-01184-3)
Supplement: Supplementary file 1 — Additional file 1. Table S1: Gene list of IPA Pathway. Table S2: DEGs with high fold change. Table S3: Antibody list for Western blotting, IHC, and IF. Table S4: ELISA kit for serum cytokines analysis. Table S5: Primer list for qRT-PCR. [file 13578_2023_1184_MOESM1_ESM.docx]

| **IPA pathway** | **-log (p-value)** | **Ratio** | **z-score** | **Molecules** |
| --- | --- | --- | --- | --- |
| Acetone Degradation I (to Methylglyoxal) | 7.9 | 0.33 | 1.90 | *Cyp4f8,Cyp4a22,Fam213b,*  *Cyp3a5,Cyp2e1,Cyp2c18,Dhrs11,Cyp2a6, Cyp4a11,Cyp2c8* |
| LPS/IL-1 Mediated Inhibition of RXR Function | 7.54 | 0.11 | -1.51 | *Abcg8,Apoe,Acsl3,Abcg5,Gstm5,*  *Il1r1,Alas1,Gstt2/Gstt2b,Cyp2a12/Cyp2a22,Nr0b2,Il1rn,Cyp3a5,Aldh3a2,Nr1i3,Cyp7a1,Gstm4,Cd14,Acsl4,Fabp4,Aldh18a1,Cyp2a6,Fabp5,Abcc,Cyp4a11,Cyp2c8* |
| Methylglyoxal Degradation III | 6.03 | 0.37 | 1.89 | *Akr7a2,Cyp4a22,Fam213b,Akr1c3,Cyp2e1,Dhrs11,Cyp4a11* |
| LXR/RXR Activation | 6 | 0.13 | 2.53 | *Abcg8,Apoe,Abcg5,Mlxipl,Msr1,Apoa2,Apoa5,Il1r1,Rxrg,Lyz,Il1rn,Ipl,Cyp7a1,Cd14,Serpina1,Hmgcr* |
| Hepatic Fibrosis / Hepatic Stellate Cell Activation | 5.28 | 0.10 | N/A | *Myh10,Col5a2,Ccr5,Vcam1,Col4a5,Ctgf,Col4a1,Il6r,Klf6,Smad7,Il1r1,Col1a2,Col1a1,Col6a3,Acta2,Cyp2e1,Pdgfra,Cd14Col3a1* |
| Fatty Acid  β-oxidation I | 4.35 | 0.22 | 1.13 | *Acaa1b,Acsl3,Acaa1,Acsl4,Ehhadh,Eci1,Hsd17b8* |
| GP6 Signaling Pathway | 3 | 0.09 | -2.31 | *Col1a2,Col1a1,Col5a2,Col4a1,Col4a5,Col6a3,Lama2,Lama3,Lamb1,Adam10,Irs2,Col3a1* |
| Triacylglycerol Degradation | 2.89 | 0.13 | 1.89 | *Ces2a,Faah,Ces1e,Ces1,Lpl,Notum,Lipe* |
| PPAR Signaling | 1.89 | 0.08 | 1.41 | *Hsp90b1,Nr0b2,Il1rn,Pdgfra,Hsp90aa1,Il1r1,Map4k4,Tab1* |
| Triacylglycerol Biosynthesis | 0.369 | 0.04 | N/A | *Lpin1,Lpin2* |

Supplemental -Table 1. Gene list of IPA Pathway

| **Symbol** | **WT-**  **CCl_4_** | | **KO-**  **CCl_4_** | **Log2FC**  **KO/WT** | | **adj p-value** | |
| --- | --- | --- | --- | --- | --- | --- | --- |
| *Eno1b* | 1107.26 | 1.43 | | -9.60 | 8.72E-03 | |  |
| *Tmc5* | 175.41 | 0.34 | | -9.01 | 3.29E-12 | |  |
| *Fkbp51* | 684.94 | 11.29 | | -5.92 | 8.47E-57 | |  |
| *A2m* | 71.17 | 1.43 | | -5.64 | 9.69E-02 | |  |
| *Mettl7a2* | 54.64 | 1.43 | | -5.25 | 2.14E-06 | |  |
| *Akr1c18* | 113.32 | 5.22 | | -4.44 | 7.47E-14 | |  |
| *Lcn2* | 9309.15 | 577.42 | | -4.01 | 2.90E-03 | |  |
| *Bhlha15* | 129.93 | 8.50 | | -3.93 | 5.61E-07 | |  |
| *Saa2* | 20064.29 | 1464.65 | | -3.78 | 5.00E-02 | |  |
| *Alpk1* | 120.53 | 9.02 | | -3.74 | 1.01E-04 | |  |
| *Prtn3* | 113.13 | 8.74 | | -3.69 | 3.70E-15 | |  |
| *Cyp7a1* | 1453.10 | 118.92 | | -3.61 | 8.59E-03 | |  |
| *Tff3* | 67.67 | 5.64 | | -3.58 | 1.26E-02 | |  |
| *Abcb1b* | 127.78 | 10.88 | | -3.55 | 5.69E-02 | |  |
| *Lox* | 54.45 | 4.95 | | -3.46 | 3.81E-03 | |  |
| *Rnaset2b* | 298.54 | 30.10 | | -3.31 | 1.24E-20 | |  |
| *Steap4* | 7051.09 | 713.74 | | -3.30 | 1.69E-02 | |  |
| *Ugt2b37* | 139.77 | 14.16 | | -3.30 | 1.63E-01 | |  |
| *Mmp12* | 278.39 | 29.94 | | -3.22 | 2.53E-03 | |  |
| *Nucb2* | 90.67 | 10.08 | | -3.17 | 6.16E-11 | |  |
| *Anln* | 54.39 | 6.27 | | -3.12 | 1.37E-02 | |  |
| *Adcy1* | 55.76 | 6.68 | | -3.06 | 2.29E-02 | |  |
| *Saa3* | 1121.34 | 138.81 | | -3.01 | 1.21E-06 | |  |
| *Cxcl14* | 311.46 | 39.25 | | -2.99 | 3.00E-02 | |  |
| *Zfp9* | 73.20 | 9.35 | | -2.97 | 1.05E-08 | |  |
| *Megf9* | 83.53 | 11.37 | | -2.88 | 3.33E-03 | |  |
| *Lpin1* | 4115.20 | 563.91 | | -2.87 | 9.73E-11 | |  |
| *Mki67* | 132.86 | 18.26 | | -2.86 | 1.21E-01 | |  |
| *AI607873* | 78.17 | 10.76 | | -2.86 | 1.78E-02 | |  |
| *Spp1* | 1595.96 | 220.01 | | -2.86 | 1.42E-04 | |  |
| *B3galt1* | 567.08 | 80.98 | | -2.81 | 1.28E-01 | |  |
| *Tiam2* | 92.19 | 13.26 | | -2.80 | 2.50E-09 | |  |
| *Ect2* | 73.79 | 11.41 | | -2.69 | 1.12E-01 | |  |
| *Myom2* | 180.80 | 28.15 | | -2.68 | 1.29E-02 | |  |
| *Pnpla3* | 81.87 | 12.98 | | -2.66 | 2.17E-01 | |  |
| *Mt1* | 3968.46 | 634.51 | | -2.64 | 9.05E-02 | |  |
| *Ckap2* | 95.23 | 15.34 | | -2.63 | 5.76E-02 | |  |
| *Saa1* | 22148.33 | 3623.77 | | -2.61 | 7.86E-02 | |  |
| *Knstrn* | 169.27 | 28.61 | | -2.56 | 1.10E-04 | |  |
| *Ccnb1* | 143.92 | 24.54 | | -2.55 | 2.59E-02 | |  |
| *Filip1l* | 67.56 | 11.78 | | -2.52 | 4.14E-03 | |  |
| *Elf3* | 57.38 | 10.17 | | -2.50 | 4.96E-02 | |  |
| *Mt2* | 3634.92 | 647.47 | | -2.49 | 4.83E-03 | |  |
| *Afp* | 108.00 | 19.40 | | -2.48 | 2.48E-02 | |  |
| *Adam8* | 159.08 | 28.79 | | -2.47 | 5.05E-05 | |  |
| *Gpnmb* | 1463.89 | 266.59 | | -2.46 | 3.22E-02 | |  |
| *Dusp8* | 104.86 | 19.42 | | -2.43 | 1.41E-02 | |  |
| *Scara5* | 453.66 | 84.06 | | -2.43 | 1.63E-02 | |  |
| *Plin4* | 191.60 | 35.61 | | -2.43 | 2.90E-07 | |  |
| *Slc39a10* | 54.20 | 10.08 | | -2.43 | 4.33E-05 | |  |
| *Tmc7* | 89.67 | 16.79 | | -2.42 | 1.86E-04 | |  |
| *Zbtb16* | 357.44 | 69.66 | | -2.36 | 1.75E-04 | |  |
| *Ckap2l* | 97.57 | 19.04 | | -2.36 | 6.20E-02 | |  |
| *Btg3* | 133.73 | 26.21 | | -2.35 | 2.11E-07 | |  |
| *Cyp3a44* | 134.50 | 26.46 | | -2.35 | 3.22E-02 | |  |
| *Atp6v0d2* | 59.44 | 11.81 | | -2.33 | 2.87E-02 | |  |
| *Atp4a* | 77.01 | 15.32 | | -2.33 | 1.12E-03 | |  |
| *Postn* | 135.55 | 27.47 | | -2.30 | 1.14E-05 | |  |
| *Hebp2* | 50.59 | 10.40 | | -2.28 | 1.03E-04 | |  |
| *Tlr13* | 75.30 | 15.49 | | -2.28 | 3.55E-06 | |  |
| *Acta2* | 106.06 | 21.83 | | -2.28 | 1.71E-07 | |  |
| *Slc10a2* | 311.89 | 64.39 | | -2.28 | 9.83E-04 | |  |
| *Tpx2* | 166.75 | 34.50 | | -2.27 | 9.23E-02 | |  |
| *Aldh18a1* | 103.17 | 21.53 | | -2.26 | 5.49E-07 | |  |
| *Itgax* | 95.22 | 20.27 | | -2.23 | 1.08E-06 | |  |
| *Cd14* | 260.77 | 55.54 | | -2.23 | 4.86E-02 | |  |
| *Ccnb2* | 137.57 | 29.41 | | -2.23 | 4.42E-04 | |  |
| *Mgp* | 88.73 | 18.98 | | -2.23 | 2.96E-02 | |  |
| *C4a* | 696.75 | 149.31 | | -2.22 | 3.86E-03 | |  |
| *Osmr* | 126.10 | 27.10 | | -2.22 | 1.17E-07 | |  |
| *Plek* | 61.36 | 13.23 | | -2.21 | 8.52E-04 | |  |
| *Fabp5* | 381.23 | 82.65 | | -2.21 | 2.28E-07 | |  |
| *Ccna2* | 149.56 | 32.74 | | -2.19 | 2.45E-02 | |  |
| *S100a8* | 122.03 | 26.72 | | -2.19 | 6.63E-02 | |  |
| *Tnc* | 99.07 | 21.75 | | -2.19 | 8.52E-05 | |  |
| *S100a9* | 278.95 | 61.52 | | -2.18 | 5.58E-02 | |  |
| *1600002H07Rik* | 1076.88 | 246.00 | | -2.13 | 5.50E-09 | |  |
| *Smc4* | 62.90 | 14.71 | | -2.10 | 2.03E-01 | |  |
| *Tm6sf1* | 56.56 | 13.26 | | -2.09 | 1.69E-04 | |  |
| *Hmgcr* | 2359.45 | 556.46 | | -2.08 | 3.88E-03 | |  |
| *Armcx3* | 184.81 | 43.76 | | -2.08 | 4.34E-08 | |  |
| *Tacc3* | 95.91 | 22.75 | | -2.08 | 1.28E-02 | |  |
| *Hsp90aa1* | 1139.04 | 270.48 | | -2.07 | 1.00E-02 | |  |
| *Il1r1* | 1076.33 | 256.96 | | -2.07 | 5.86E-03 | |  |
| *Prc1* | 67.05 | 16.05 | | -2.06 | 2.38E-02 | |  |
| *Arhgap11a* | 72.40 | 17.35 | | -2.06 | 1.84E-01 | |  |
| *Cdkn3* | 52.09 | 12.49 | | -2.06 | 6.93E-04 | |  |
| *C5ar1* | 76.09 | 18.29 | | -2.06 | 2.65E-05 | |  |
| *C730036E19Rik* | 144.58 | 34.99 | | -2.05 | 6.29E-02 | |  |
| *Atp6v0c* | 1452.85 | 355.42 | | -2.03 | 2.12E-08 | |  |
| *Icosl* | 50.30 | 12.40 | | -2.02 | 5.04E-04 | |  |
| *Akap2* | 145.26 | 35.88 | | -2.02 | 4.53E-07 | |  |
| *Rassf2* | 97.94 | 24.29 | | -2.01 | 7.06E-06 | |  |
| *Anxa1* | 108.99 | 27.15 | | -2.00 | 1.73E-03 | |  |
| *Dbf4* | 69.89 | 17.45 | | -2.00 | 4.96E-03 | |  |
| *Atp6v0c-ps2* | 4.29 | 1416.93 | | 8.37 | 2.14E-91 | |  |
| *Cyp2b9* | 1.65 | 52.97 | | 5.01 | 4.78E-02 | |  |
| *Cyp2c39* | 18.97 | 432.18 | | 4.51 | 1.46E-08 | |  |
| *0610010B08Rik* | 2.96 | 58.53 | | 4.30 | 1.19E-01 | |  |
| *Cyp4a14* | 213.83 | 4087.58 | | 4.26 | 1.17E-01 | |  |
| *Clec2h* | 6.26 | 104.70 | | 4.06 | 7.11E-05 | |  |
| *Cyp2a5* | 144.45 | 2255.74 | | 3.96 | 5.92E-04 | |  |
| *Nr4a1* | 25.11 | 265.08 | | 3.40 | 1.71E-03 | |  |
| *Cyp2a4* | 7.09 | 73.37 | | 3.37 | 1.28E-02 | |  |
| *Elovl3* | 63.39 | 638.51 | | 3.33 | 1.89E-01 | |  |
| *Syt3* | 12.14 | 100.15 | | 3.04 | 5.52E-11 | |  |
| *Gm10653* | 13.77 | 110.03 | | 3.00 | 1.94E-11 | |  |
| *Cyp2c38* | 11.49 | 86.23 | | 2.91 | 2.50E-02 | |  |
| *Hamp2* | 105.66 | 760.42 | | 2.85 | 4.74E-02 | |  |
| *Cebpe* | 10.15 | 71.66 | | 2.82 | 2.14E-06 | |  |
| *Cyr61* | 48.75 | 339.21 | | 2.80 | 1.69E-07 | |  |
| *Ppp1r3g* | 8.86 | 59.17 | | 2.74 | 3.62E-07 | |  |
| *Abcg8* | 198.99 | 1224.03 | | 2.62 | 5.27E-09 | |  |
| *Park2* | 12.20 | 73.38 | | 2.59 | 1.48E-07 | |  |
| *Slc1a2* | 18.21 | 98.72 | | 2.44 | 2.38E-03 | |  |
| *Gm4956* | 16.97 | 90.58 | | 2.42 | 1.48E-07 | |  |
| *Gsta2* | 85.30 | 454.13 | | 2.41 | 1.30E-10 | |  |
| *Nr0b2* | 124.57 | 637.89 | | 2.36 | 9.63E-07 | |  |
| *Bik* | 11.38 | 57.86 | | 2.35 | 5.58E-02 | |  |
| *Spry4* | 28.20 | 140.76 | | 2.32 | 8.97E-09 | |  |
| *Etnppl* | 198.66 | 960.46 | | 2.27 | 5.24E-02 | |  |
| *Dbp* | 76.56 | 360.12 | | 2.23 | 1.78E-01 | |  |
| *Marcksl1-ps4* | 13.41 | 63.03 | | 2.23 | 2.41E-05 | |  |
| *8430408G22Rik* | 356.58 | 1647.59 | | 2.21 | 4.39E-02 | |  |
| *Id2* | 246.94 | 1136.30 | | 2.20 | 5.43E-13 | |  |
| *Dpy19l3* | 39.65 | 181.16 | | 2.19 | 7.74E-09 | |  |
| *Pde4b* | 31.20 | 141.17 | | 2.18 | 3.73E-03 | |  |
| *S100g* | 12.44 | 53.58 | | 2.11 | 1.27E-04 | |  |
| *D630039A03Rik* | 53.00 | 227.50 | | 2.10 | 2.59E-05 | |  |
| *Trib3* | 45.96 | 194.16 | | 2.08 | 6.44E-03 | |  |
| *Bmf* | 62.55 | 261.51 | | 2.06 | 9.58E-06 | |  |
| *Gclc* | 896.86 | 3737.78 | | 2.06 | 5.49E-12 | |  |
| *Slc25a25* | 1223.06 | 4942.82 | | 2.01 | 2.90E-05 | |  |
| *Rnf43* | 120.37 | 482.50 | | 2.00 | 1.08E-09 | |  |

Supplemental Table 2: DEGs with high fold change

| **Antibody** | **Company** | **Species** | **WB** | **IF/****IHC** |
| --- | --- | --- | --- | --- |
| Alexa Flour® 488-conjugated donkey anti-Goat IgG | Abcam | donkey |  | 1:500 |
| Alexa Flour® 488-conjugated donkey anti-Mouse IgG | Abcam | donkey |  | 1:500 |
| Alexa Flour® 488-conjugated donkey anti-Rabbit IgG | Abcam | donkey |  | 1:500 |
| Alexa Flour® 594-conjugated donkey anti-Mouse IgG | Abcam | donkey |  | 1:500 |
| AKT | Cell Signaling Technology | rabbit | 1:1000 |  |
| Collagen I | Abcam | rabbit |  | 1:100 |
| COX IV | Cell Signaling Technology | rabbit | 1:1000 |  |
| CTGF | Abcam | rabbit |  | 1:200 |
| DRP1 | Abcam | rabbit | 1:1000 |  |
| FKBP51 | Santa Cruz Biotechnology | goat | 1:1000 | 1:200 |
| GAPDH | Santa Cruz Biotechnology | rabbit | 1:5000 |  |
| LC3B | Cell Signaling Technology | rabbit | 1:1000 | 1:100 |
| P62 | MBL | rabbit | 1:1000 |  |
| pAKT | Cell Signaling Technology | rabbit | 1:1000 |  |
| Parkin | Biolegend | mouse | 1:1000 | 1:200 |
| pDRP1 | Cell Signaling Technology | rabbit | 1:1000 |  |
| PINK1 | Abcam | rabbit | 1:1000 |  |
| TIMP1 | Santa Cruz | rabbit | 1:1000 | 1:100 |
| VDAC | Cell Signaling Technology | rabbit | 1:1000 |  |
| α-SMA | Sigma | rabbit | 1:4000 | 1:100 |

Supplemental Table 3. Antibody list for Western blotting, IHC, and IF.

| **ELISA Kit** | **Cat.** |
| --- | --- |
| Mouse TGF-β1 ELISA Kit | XFM1292A |
| Mouse IFN-γ ELISA Kit | XFM1045A |
| Mouse GC ELISA Kit | XFM0215A |
| Mouse NF-κB ELISA Kit | XFM3188A |
| Mouse IL-6 ELISA Kit | XFM1612A |
| Mouse IL-10 ELISA Kit | XFM1074A |
| Mouse TNF-α ELISA Kit | XFM1753A |
| Mouse FGF ELISA Kit | XFM1043A |

Supplemental Table 4. ELISA kit for serum cytokines analysis.

| Gene | Forward | Reverse |
| --- | --- | --- |
| *Fkbp51* | AATCAAACGGAAAGGCGAGGGATAC | CCAATCGGAATGTCGTGGTCTTCTC |
| *Park2* | TTTTCATCTACTGCAAAGGCCC | TCACCACTCATCCGGTTTGG |
| *Gapdh* | CATCACTGCCACCCAGAAGACTG | ATGCCAGTGAGCTTCCCGTTCAG |

Supplemental Table 5. Primer list for qRT-PCR.
