# Supplementary material for: Knocking out Fkbp51 decreases CCl4-induced liver injury through enhancement of mitochondrial function and Parkin activity
Source: Cell Biosci. 2024 Jan 2;14:1. doi: 10.1186/s13578-023-01184-3 (PMC10763032; doi:10.1186/s13578-023-01184-3)
Supplement: Supplementary file 2 — Additional file 2. Fig. S1: Immunohistochemical comparisons of Control and CCl4-treated Fkbp51 KO and WT liver sections. Expression patterns of A Collagen I, B CTGF, and C α-SMA are all higher in the livers of CCl4 treated WT mice than in KO mice. Key: WT, wild type; KO, Fkbp51 KO. Fig. S2: Serum analyses of Control and CCl4-treated Fkbp51 KO and WT were performed to determine the concentrations of A IFN-γ, B IL-6, C TGF-β1, D NFκB, E IL-10, F TNF-α, G GC, and H FGF. Graphs represent mean values ± SEM from 6 mice for each group. p values were determined by two-way ANOVA with the statistical significance labeled as follows: *as p < 0.05, ** as p < 0.01 and **** as p < 0.0001. Key: WT, wild type; KO, Fkbp51 KO. Fig. S3: A IPA pathway analysis identified disease and development, physiological system and development, signaling pathways, and toxicity-related changes as different between KO and WT after CCl4 injury. B Go analysis further suggested the enrichment of biological process, cellular component, and molecular function. The top 10 relevant pathways are included in the bar graph. Fig. S4: The quantification for Fig. 4i, j. A Increased cololization of Parkin/MitoTracker in KO MEFs. B The increased relative normalized MFI of Parkin per cell in KO MEFs. C Colocalization between Flag-FKBP51/Parkin, Parkin/Flag-FKBP51, Flag-FKBP51/MitoTracker and Parkin/MitoTracker. Graph represents mean values ± SEM from 3 independent experiments. p values were determined by student’s unpaired t-test (A, B) with the statistical significance labeled as follows: *** as p < 0.001, **** as p < 0.0001. Key: WT, wild type; KO, Fkbp51 KO. Fig. S5: Representative EM photomicrographs demonstrate that KO has less ER expansion than WT, and KO liver mitochondria are lighter in appearance than WT mitochondria after CCl4 treatment. Fig. S6: The quantification for Fig. 8a. A, B The relative normalized MFI of Mitotracker and LC3B per cell from both genotype under DMSO or CCCP treatment. C Colocalizati [file 13578_2023_1184_MOESM2_ESM.pptx]

## Slide 1
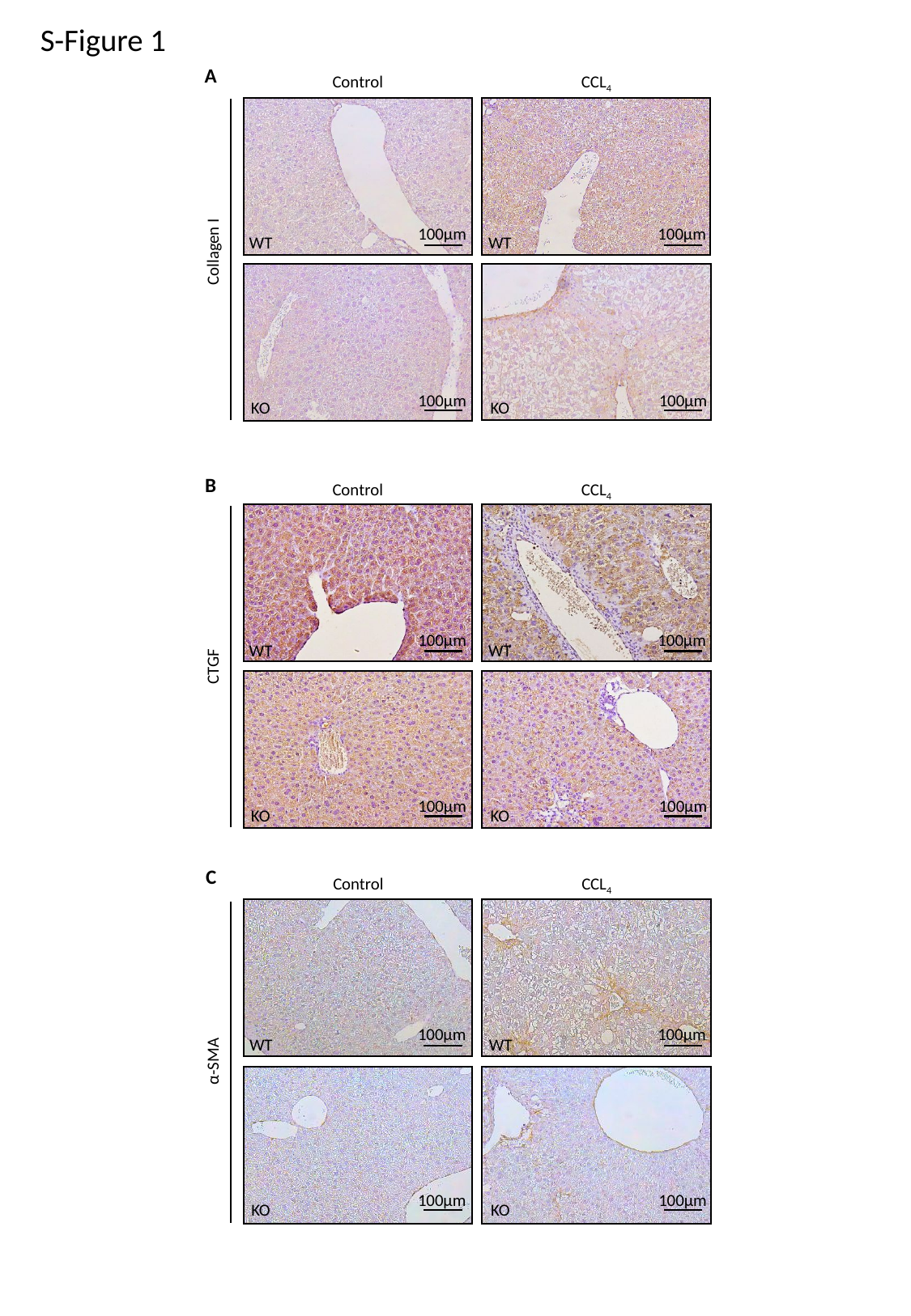

S-Figure 1
A
Control
CCL4
100µm
100µm
WT
WT
Collagen I
100µm
100µm
KO
KO
B
Control
CCL4
100µm
100µm
WT
WT
CTGF
100µm
100µm
KO
KO
C
Control
CCL4
100µm
100µm
WT
WT
α-SMA
100µm
100µm
KO
KO

## Slide 2
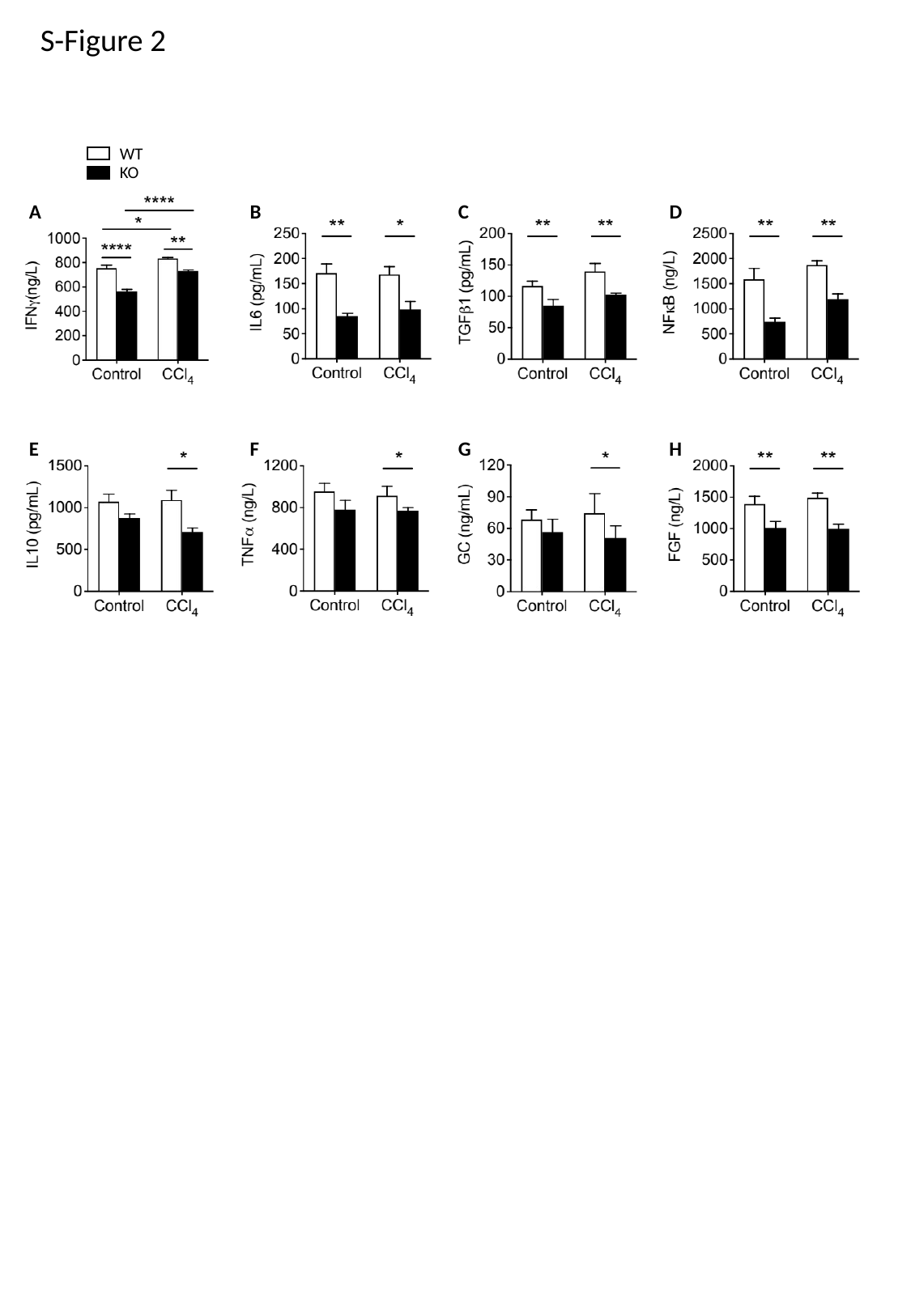

S-Figure 2
WT
KO
A
B
C
D
E
F
G
H

## Slide 3
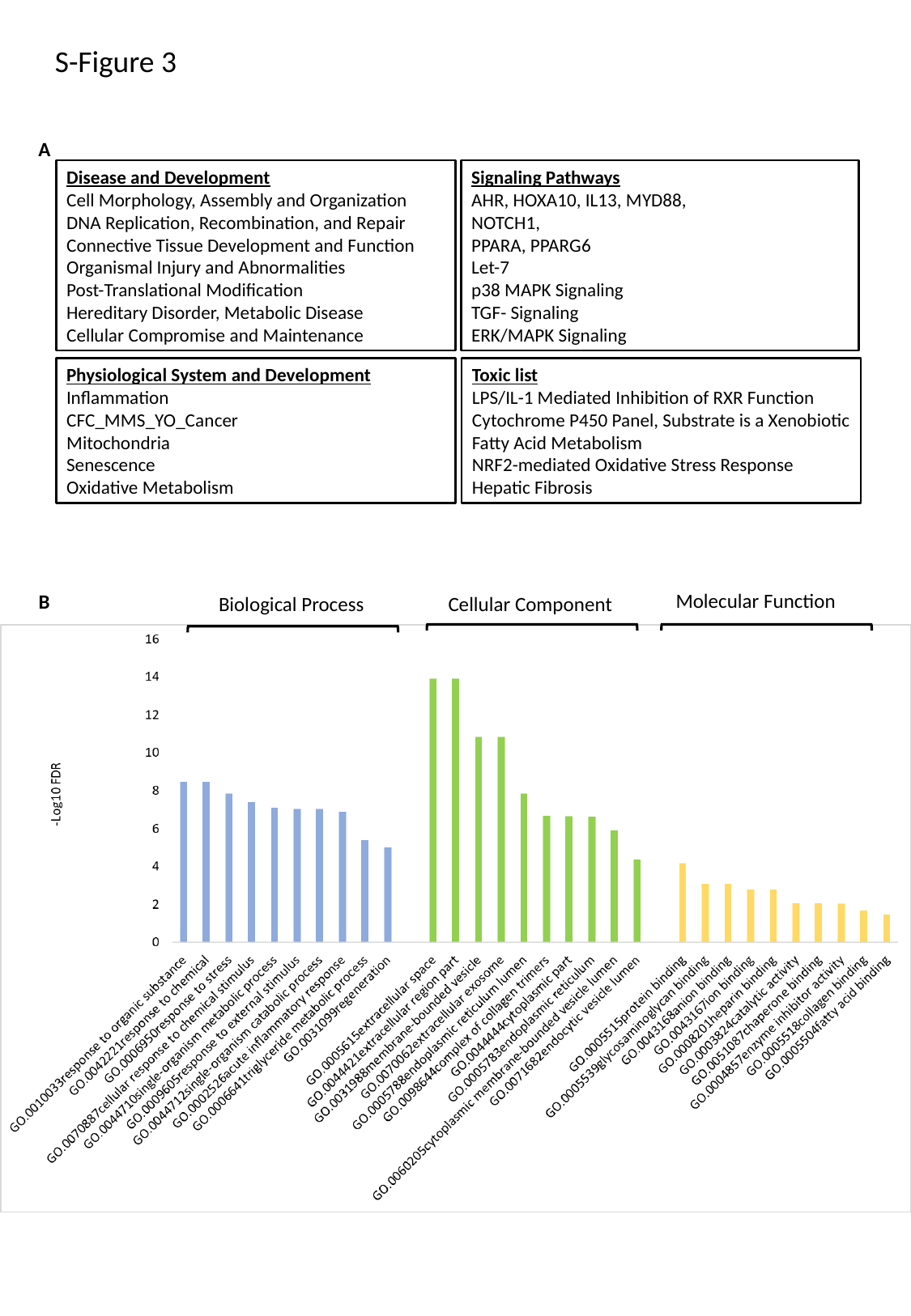

S-Figure 3
A
Disease and Development
Cell Morphology, Assembly and Organization
DNA Replication, Recombination, and Repair
Connective Tissue Development and Function
Organismal Injury and Abnormalities
Post-Translational Modification
Hereditary Disorder, Metabolic Disease
Cellular Compromise and Maintenance
Signaling Pathways
AHR, HOXA10, IL13, MYD88,
NOTCH1,
PPARA, PPARG6
Let-7
p38 MAPK Signaling
TGF- Signaling
ERK/MAPK Signaling
Physiological System and Development
Inflammation
CFC_MMS_YO_Cancer
Mitochondria
Senescence
Oxidative Metabolism
Toxic list
LPS/IL-1 Mediated Inhibition of RXR Function
Cytochrome P450 Panel, Substrate is a Xenobiotic
Fatty Acid Metabolism
NRF2-mediated Oxidative Stress Response
Hepatic Fibrosis
Molecular Function
Biological Process
Cellular Component
B

## Slide 4
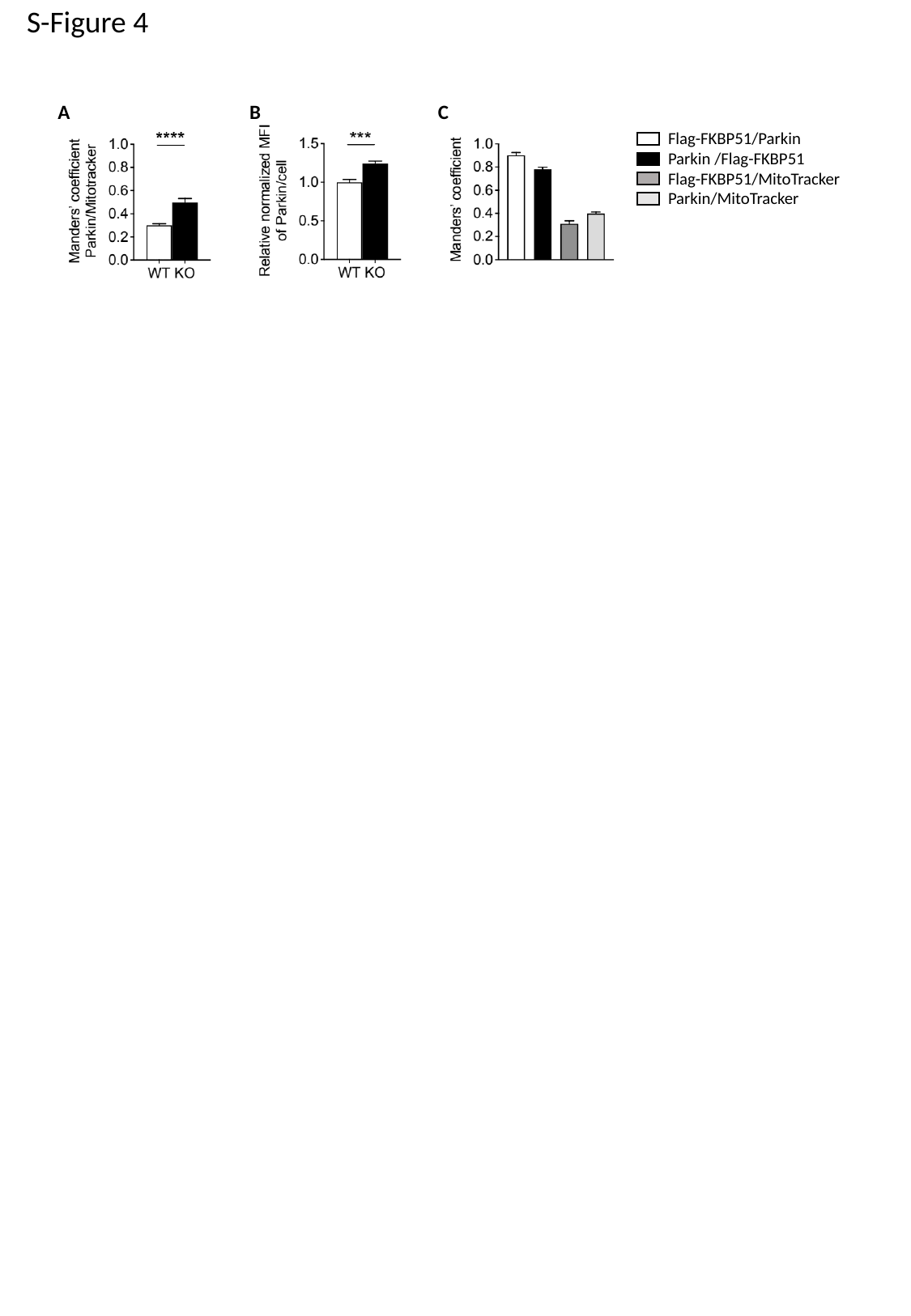

S-Figure 4
A
B
C
Flag-FKBP51/Parkin
Parkin /Flag-FKBP51
Flag-FKBP51/MitoTracker
Parkin/MitoTracker

## Slide 5
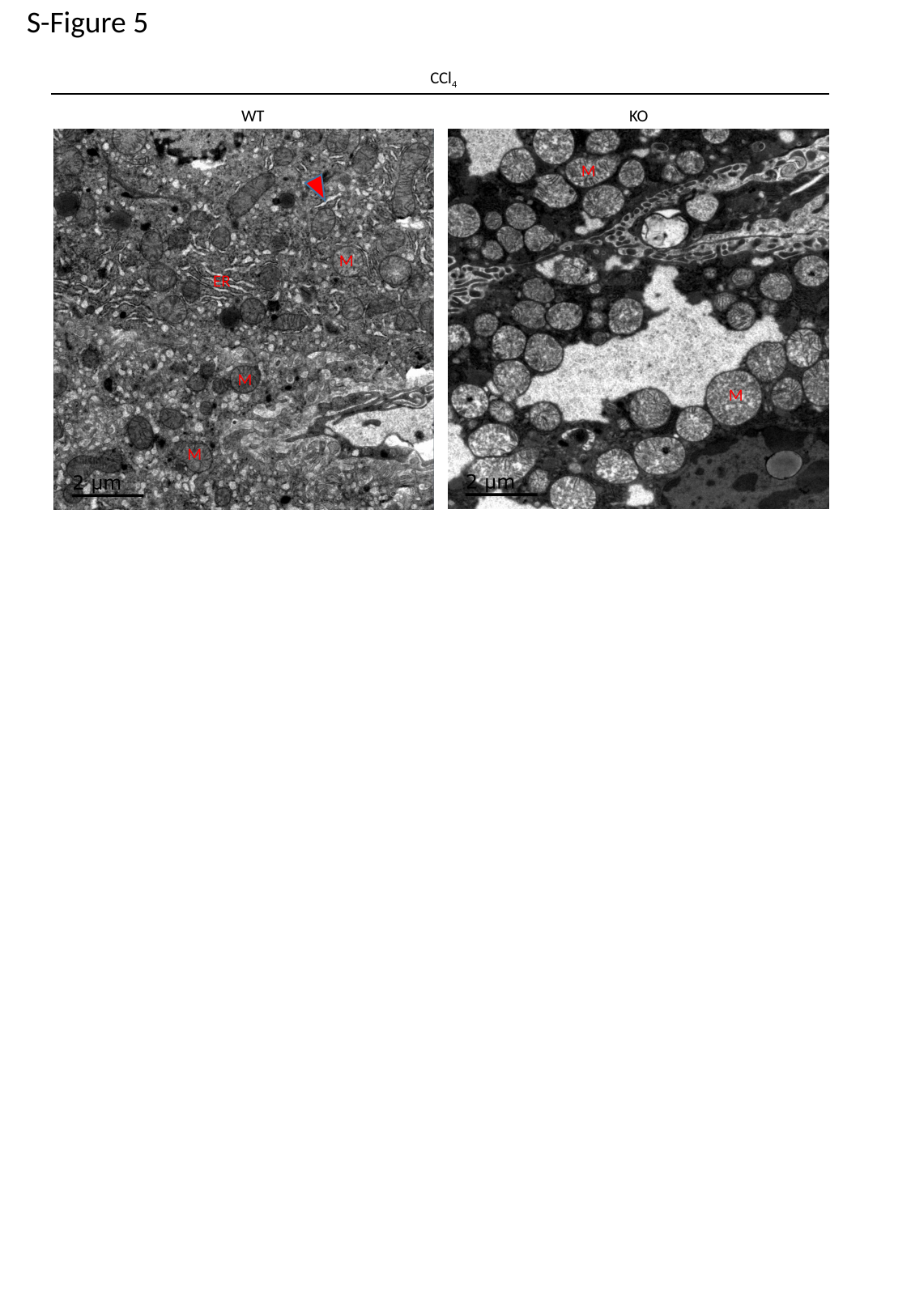

S-Figure 5
CCl4
WT
KO
M
M
ER
M
M
M

## Slide 6
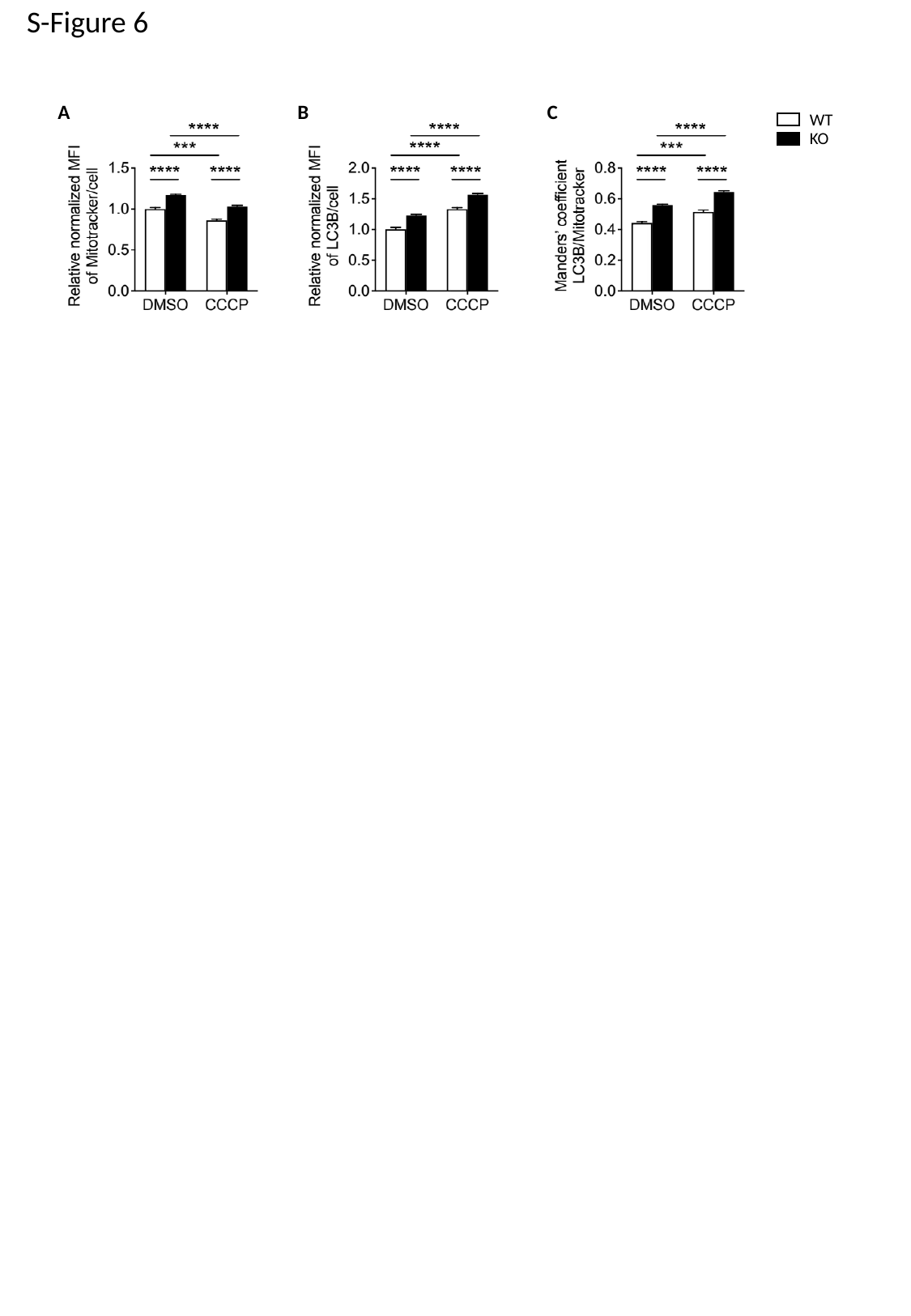

S-Figure 6
A
B
C
WT
KO

## Slide 7
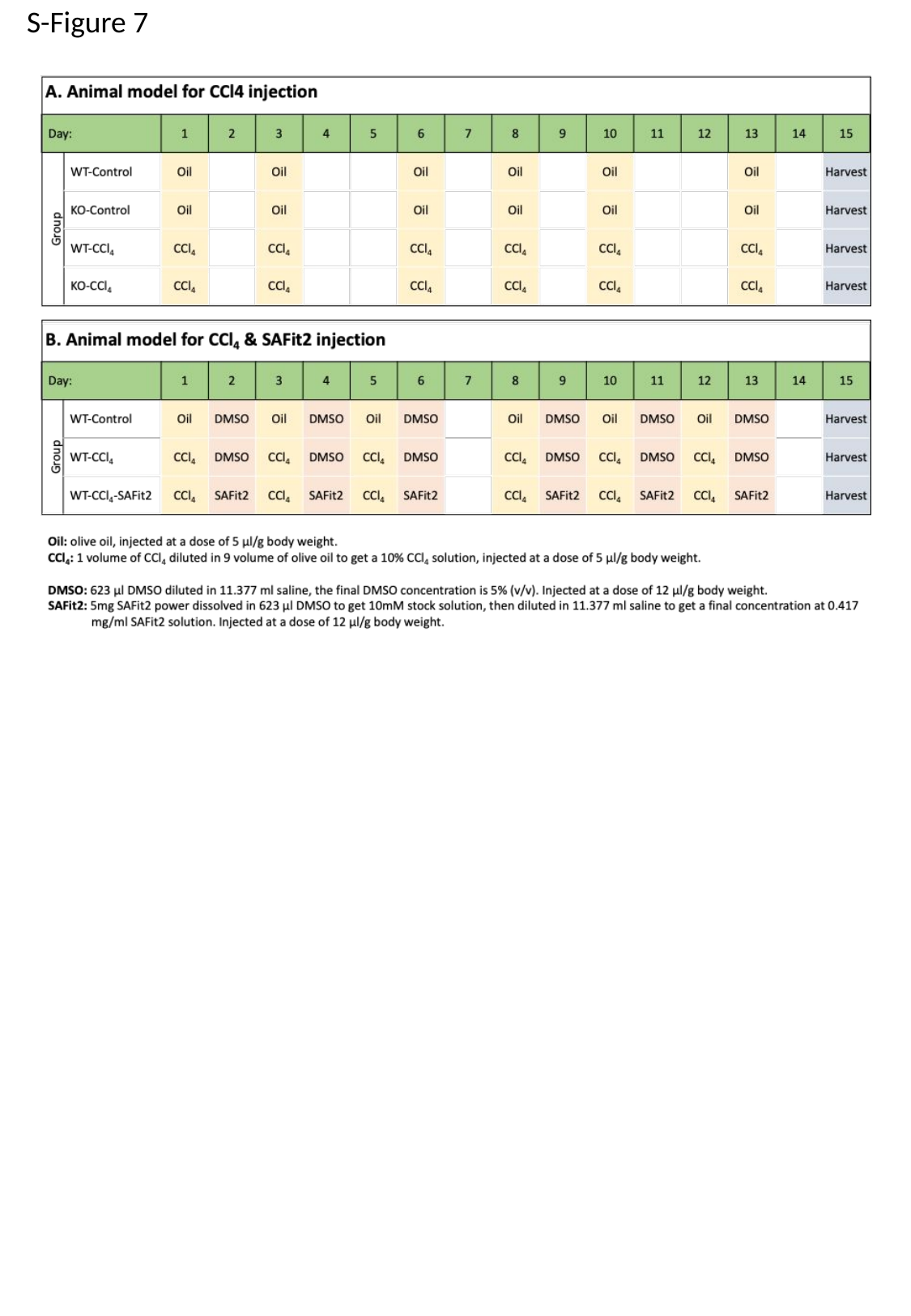

S-Figure 7
